# Real-world data: a comprehensive literature review on the barriers, challenges, and opportunities associated with their inclusion in the health technology assessment process

**DOI:** 10.3389/jpps.2024.12302

**Published:** 2024-02-28

**Authors:** Konstantinos Zisis, Elpida Pavi, Mary Geitona, Kostas Athanasakis

**Affiliations:** ^1^ Laboratory for Health Technology Assessment (LabHTA), Department of Public Health Policy, School of Public Health, University of West Attica, Athens, Greece; ^2^ Institute for Health Economics, Athens, Greece; ^3^ Department of Social and Educational Policy, Faculty of Social and Political Sciences, University of Peloponnese, Corinth, Greece

**Keywords:** real-world data, real-world evidence, health technology assessment, acceptance, barriers, challenges

## Abstract

**Objective:** This review aimed to assess the current use and acceptance of real-world data (RWD) and real-world evidence (RWE) in health technology assessment (HTA) process. It additionally aimed to discern stakeholders’ viewpoints concerning RWD and RWE in HTA and illuminate the obstacles, difficulties, prospects, and consequences associated with the incorporation of RWD and RWE into the realm of HTA.

**Methods:** A comprehensive PRISMA-based systematic review was performed in July 2022 in PubMed/Medline, Scopus, IDEAS-RePEc, International HTA database, and Centre for Reviews and Dissemination with *ad hoc* supplementary search in Google Scholar and international organization websites. The review included pre-determined inclusion criteria while the selection of eligible studies, the data extraction process and quality assessment were carried out using standardized and transparent methods.

**Results:** Twenty-nine (*n* = 29) studies were included in the review out of 2,115 studies identified by the search strategy. In various global contexts, disparities in RWD utilization were evident, with randomized controlled trials (RCTs) serving as the primary evidence source. RWD and RWE played pivotal roles, surpassing relative effectiveness assessments (REAs) and significantly influencing decision-making and cost-effectiveness analyses. Identified challenges impeding RWD integration into HTA encompassed limited local data access, complexities in non-randomized trial design, data quality, privacy, and fragmentation. Addressing these is imperative for optimal RWD utilization. Incorporating RWD/RWE in HTA yields multifaceted advantages, enhancing understanding of treatment efficacy, resource utilization, and cost analysis, particularly via patient registries. RWE complements assessments of advanced therapy medicinal products (ATMPs) and rare diseases. Local data utilization strengthens HTA, bridging gaps when RCT data is lacking. RWD aids medical device decision-making, cancer drug reassessment, and indirect treatment comparisons. Challenges include data availability, stakeholder acceptance, expertise, and privacy. However, standardization, training, collaboration, and guidance can surmount these barriers, fostering enhanced RWD utilization in HTA.

**Conclusion:** This study highlights the intricate global landscape of RWD and RWE acceptance in HTA. Recognizing regional nuances, addressing methodological challenges, and promoting collaboration are pivotal, among others, for leveraging RWD and RWE effectively in healthcare decision-making.

## Introduction

RWE and RWD are increasingly used for evaluating health technologies to inform decision-making in the healthcare sector. RWD refers to data related to patient health status and/or the delivery of healthcare that are routinely collected from various sources outside of traditional clinical trial settings. RWE refers to data generated from RWD and it’s actually the clinical evidence about the usage, benefits, and risks of medical products, which is derived from the analysis of RWD. The evidence derives from sources such as electronic health records, claims data, product or disease registries, pragmatic trials, and data generated by patients (patient-reported outcomes) as well as digital health technologies, among others [1, 2]. RWE can provide a more comprehensive and representative picture of how treatments and interventions work in real-world conditions, beyond the controlled environment of clinical trials. The role of RWE is undergoing continuous development and broadening while has gained prominence in healthcare decision-making, particularly during the COVID-19 pandemic [3]. While RCTs are still considered the benchmark for assessing the effectiveness of treatments including new cancer treatments, there is a growing consensus that relying solely on RCTs may not provide comprehensive solutions to all pertinent clinical or research inquiries and RWE can contribute in advancing decisions by providing complementary evidence [4].

In a general context, the advantages of using RWD in patient care are to:

• Evaluate the effectiveness and safety of treatments and interventions in real-world populations and environments provides a more holistic view of patient health and care outcomes, as data is derived from routine clinical care rather than controlled settings.
• generate data on subpopulations that may be underrepresented in clinical trials by capturing a wider range of patient populations and health conditions, including underrepresented groups, and identify rare or long-term adverse events that may not be captured in clinical trials.
• monitor the safety and efficacy of new treatments or interventions in real-world settings, beyond the limited scope of clinical trials [5].

The utilization of RWD and the generation of RWE hold immense promise for transforming healthcare decision-making. However, there are also challenges associated with the use of RWD, including issues related to inconsistent data quality, comparability and bias (subject to bias and measurement errors, both random and non-random) [6], as well as the need for appropriate statistical methods and analytical frameworks. Such challenges among others, are the following:

⁃ Data Quality and Consistency: RWD originates from various sources in the real-world healthcare ecosystem, including electronic health records, claims databases, and patient registries. Consequently, data quality can be inconsistent due to differences in data collection methodologies and standards across healthcare institutions. Incomplete, inaccurate, or missing data can lead to flawed analyses and unreliable conclusions. Furthermore, the diverse nature of RWD sources means that data may vary in terms of completeness, timeliness, and relevance.
⁃ Bias and Measurement Errors: RWD is inherently subject to bias and measurement errors, which can emanate from several sources. Selection bias can occur when certain patient populations are overrepresented or underrepresented in the data due to factors such as healthcare seeking behavior or data collection practices. Information bias may arise from discrepancies in the way data is recorded or measured, leading to inaccuracies. Additionally, non-random error can be introduced through factors like data entry mistakes, misclassification of variables, or systematic differences in data collection across institutions. These biases and errors can skew RWE findings, potentially leading to misleading conclusions about the safety and effectiveness of medical interventions [7].

Considering the formidable challenges inherent in the field, it is noteworthy that the prominence of RWE in shaping healthcare decision-making continues to ascend and the importance of RWE in healthcare decision-making is growing. Regulatory agencies such as the U.S. Food and Drug Administration (FDA) recognize its potential and have issued guidance on its use in regulatory decision-making. These guidelines provide a structured framework for how RWE can be employed to support various stages of drug development and post-market surveillance. For example, the FDA has issued guidance on the use of RWE in regulatory decision-making [8], while the Institute for Clinical and Economic Review (ICER) has developed a framework [9] for integrating RWE into coverage decisions and acknowledges the value of RWE in evaluating the real-world effectiveness and cost-effectiveness of medical interventions, particularly in comparison to traditional clinical trial evidence. While RWD is progressively attaining prominence in influencing healthcare decision-making, it remains a subject of discernible complexity and resistance within the healthcare milieu.

Based on the above, the objective of the study is to investigate the integration of real-world data and real-world evidence in health technology assessment process around the world. In particular, the aim of this systematic review was to: a) assess the current utilization and level of acceptance of RWD and RWE in the HTA process, shedding light on their prevalence and applications; b) systematically prioritize and examine the barriers, challenges, opportunities, and potential implications that arise from the integration of evidence derived from RWD and RWE within the HTA process. This includes a comprehensive analysis of factors influencing successful integration; c) explore and synthesize stakeholders’ perspectives, regarding the incorporation of RWD and RWE in the HTA process.

## Materials and methods

Considering the above objective, the research questions defined for this review were the following:

➢ Is the utilization and acceptance of RWD and RWE prevalent in the HTA process?
➢ What are the barriers, challenges, potential benefits and feasibilities, as well as opportunities presented by the integration of RWD into the HTA process?
➢ What are the viewpoints and declarations of stakeholders concerning to RWD and RWE in the HTA process?

No formal protocol was established or registered for this systematic review.

### Study design, inclusion and exclusion criteria

A PRISMA-based systematic review [10, 11] was conducted to identify articles assessed by the researchers, employing inclusion criteria to ascertain study eligibility aligned with the review’s objectives. The search strategy, as detailed in *Search strategy* section and Supplementary Appendix S1, was utilized to encompass these criteria.

Inclusion criteria were as follows:

➢ Population: No restrictions were imposed on populations, and studies from diverse populations worldwide, including sub-populations, were considered eligible for inclusion.
➢ Intervention: RWD and evidence derived from the use and analysis of RWD.
➢ Comparator: No comparator.
➢ Outcomes: Data on the current use of RWD/RWE in HTA, barriers, challenges, weaknesses in their integration in the process, opportunities and stakeholders’ regarding the use of RWD/RWE in the HTA process were included. To systematically conduct the above outcome criteria search, a meticulous process was employed for the formulation of search terms. This involved a thorough review of current and relevant literature, ensuring alignment with the latest advancements and key concepts within the field to inform the selection of search terms. The carefully chosen search terms, detailed in brief within *Search strategy* section and provided in detail within Supplementary Appendix S1, were derived from this comprehensive review.
➢ Types of studies: All types of studies, such as reviews, policy texts, primary research, RCTs and qualitative research studies. This approach was adopted to ensure a thorough exploration of the subject matter, capturing both empirical data from primary studies and synthesized knowledge from reviews.
➢ Language: Studies written in English.
➢ Timeline: No time restrictions were specified for the publication of studies and policy reports.

The exclusion criteria for studies in this analysis were as follows:

➢ Study Types: Abstracts (oral and posters) that did not include at least one of the above outcome criteria.
➢ Language: Studies in languages other than English.

### Search strategy

The search strategy was meticulously designed to ensure both breadth and inclusivity. Key terms such as “accept,” “use,” “barriers,” “health technology assessment,” “real-world data,” “real-world evidence,” “opportunities,” and “stakeholders” were central to our search strategy, aiming to cast a wide net and capture a diverse range of perspectives.

The detailed search strategy, which was performed on July 2022, is provided in Supplementary Appendix S1.

Search strategy was implemented to multiple databases and particularly: PubMed/Medline, Scopus, IDEAS-RePEc, International HTA database, Centre for Reviews and Dissemination. In addition, supplementary *ad hoc* searches for relevant information were performed on Google Scholar, as well as various international organizations such as the World Health Organization (WHO) and Organisation for Economic Co-operation and Development (OECD), and specific health technology assessment organizations such as National Institute for Health and Care Excellence (NICE), Haute Autorité de santé (HAS), and Institute for Clinical & Economic Review (ICER) to identify relevant texts and references related to the study objectives.

### Study selection methods

The literature discovered through the search was archived in a bibliographic database (EndNote), with duplicate entries subsequently removed. A pilot training check process was conducted initially to ensure consistency in selection and identify areas for modifications in the inclusion criteria to provide a more comprehensive and explicit list of study types that would be considered eligible for this review. Two researchers independently checked a random sample of approximately fifty (50) titles and abstracts for eligibility, and a high level of agreement was achieved which indicates that the two researchers largely agreed on whether each of these documents met the inclusion criteria established for the study. After this, a single researcher checked the remaining titles and abstracts for eligibility. Later, the studies resulting from the removal of duplicate entries were uploaded into Abstrackr [12], a specialized software developed by Brown University and the Center for Evidence Synthesis in Health. All abstracts were examined, and full-text documents were retrieved for the files that were flagged for inclusion. The retrieved articles were then analyzed in detail based on the full text. Quality control measures, including periodic checks and inter-rater reliability assessments, were implemented to ensure the accuracy and consistency of the study selection process. This methodological approach, encompassing both manual assessment and the use of specialized software, was designed to enhance the accuracy, consistency, and transparency of the study selection process.

### Data extraction and synthesis methods

The study data was meticulously extracted and organized into four tables, a process undertaken to streamline and enhance the subsequent analysis and synthesis of the information. The design of these tables was thoughtfully structured to systematically capture pertinent information derived from the selected studies. Supplementary Table S1 contained details relevant to the characteristics including author, year of study, country, study type, objectives, health technology studied, population and therapeutic category, and subcategory of real-world data. Supplementary Table S2 was dedicated to encompassing data concerning the contemporary utilization and reception of RWD and RWE. In contrast, Supplementary Table S3 comprehensively addressed the hurdles, challenges, and complexities encountered when integrating RWD-RWE into HTA. Meanwhile, Supplementary Table S4 was designed to encompass the potential advantages, opportunities, and viability associated with the adoption of RWD-RWE within the realm of HTA. To ensure consistency and pinpoint any potential adjustments required for the data extraction model, two researchers initially conducted an independent pilot test on a random sample of ten (10) studies. During this process, an appropriate level of agreement was observed, denoting that there was a satisfactory degree of consensus or concurrence among the researchers involved in the extraction of data from the selected studies. The extraction of the remaining studies was conducted by a primary researcher, supported by a secondary researcher who remained readily available to offer assistance in clarifying information or in situations where the primary researcher encountered challenges or uncertainties during the extraction process.

### Appraisal of methodological quality

The methodological quality of the studies included in this review was assessed using several critical appraisal tools, namely, the Critical Appraisal Skills Programme (CASP) tool for qualitative research [13], the Joanna Briggs Institute (JBI) checklist for systematic reviews and evidence syntheses [14], and Joanna Briggs Institute (JBI) checklist for text and opinions [15]. Each tool evaluated different aspects of study quality by one reviewer, including the study design, data collection methods, data analysis, and reporting of results. For the assessment of each study done using the CASP tool, the reviewer assessed the quality of the study design, data collection methods, data analysis, and interpretation of findings. The Critical Appraisal Skills Programme (CASP) tool is the most used tool for quality appraisal in health-related qualitative evidence syntheses [16]. Meanwhile, the JBI checklist was used to evaluate the relevance of the studies to the review question, study design, sample size, data collection methods, data analysis, and reporting of results.

Quality appraisal, in detail, of eligible studies can be found in the Supplementary Appendix S2. In the overarching context, it is pertinent to elucidate that the quality of the incorporated studies exhibits a discernible spectrum, wherein, a number of studies may be aptly delineated as demonstrating a standard of moderate quality, while the preponderance of the corpus can be distinguished as manifesting a commendable standard of good quality.

## Results

During the search process, a total of 2,115 studies were identified based on the pre-specified selection criteria after removing duplicates (*n* = 50). Among these, 137 studies were selected for inclusion after title and abstract review. Full-text versions of all studies were obtained, with the exception of eleven studies whose authors did not respond to the request of their manuscript since were also not available in the literature. Following a thorough examination of the complete texts, 108 studies were excluded due inadequate data (*n* = 80), oral/poster presentations without much data (*n* = 14), non-availability of full-text (*n* = 11) and non-English manuscripts (*n* = 3). Eventually, 29 studies (Supplementary Table S1) out of the 137 met the inclusion criteria and were considered eligible for analysis.

**FIGURE 1:**
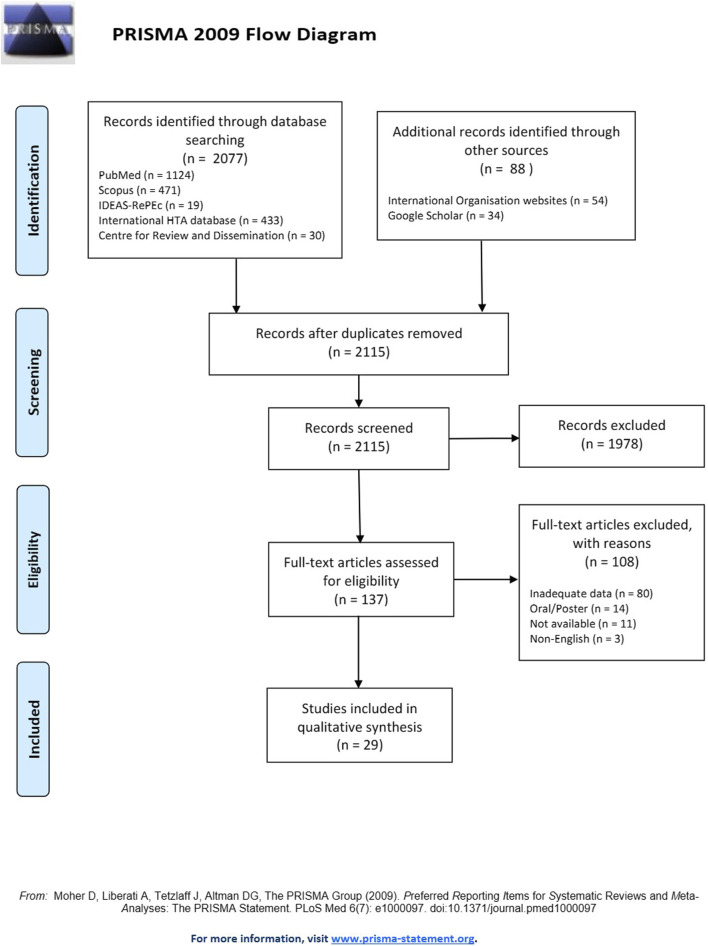
PRISMA flow chart of the search strategy.

Figure 1 illustrates the study selection process in accordance with the PRISMA flow diagram.

### Description of study characteristics

Overall, 29 studies (Supplementary Table S1) were included in this review, among which, most of them were referring to European countries. Most of these 29 studies were referring to multiple countries within their analyses while few of them assessed information related to RWD/RWE for HTA in continents. In particular, England (*n* = 6), Germany (*n* = 5), United Kingdom (*n* = 4), Sweden (*n* = 4), Netherlands (*n* = 3), Scotland (*n* = 3), France (*n* = 3), Norway (*n* = 2), Italy (*n* = 2), Spain (*n* = 1), Austria (*n* = 1), Denmark (*n* = 1), and Belgium (*n* = 1). In addition, Europe was referred in three studies, while one study included European Union countries and another one study included Central and Eastern Europe. Several included studies referred to North America, and particularly United States (*n* = 2) and Canada (*n* = 2) and one study referred to South America countries and particularly to Argentina, Brazil, Colombia, and Chile. The review also included one study referring to Asian countries (Bhutan, China, India, Indonesia, Japan, Malaysia, Philippines, Singapore, South Korea, Taiwan, Thailand). Middle East and North Africa [MENA] (*n* = 1) as well as Saudi Arabia (*n* = 1) were part of the final studies while international scope was referred in five studies.

### Current use and acceptance of real-world data/real-world evidence in HTA

The acceptance and utilization of RWD and RWE vary across HTA organizations and countries. Prevailing preferences for RCTs and systematic reviews were evident in various studies, with RWD from observational studies considered when RCTs are unavailable [17, 18]. Stakeholders generally prioritize RCTs [19, 20], including in the context of Next-Generation Sequencing technologies [21]. Notable discrepancies are observed in Saudi Arabia, with some stakeholders expressing skepticism about the reliability of RWE compared to outcomes derived from RCTs [22]. However, Austrian, French, and English HTA organizations recognize the value of observational studies, especially for biomarker assessments, diagnostic testing accuracy data, and situations where RCTs are non-feasible [23]. They also consider data from all non-randomized controlled trials (non-RCTs), particularly for medical devices assessment [24]. In Asian countries, there is a widespread positive inclination toward embracing and utilizing RWD and RWE by HTAs for assessing clinical effectiveness and reimbursing technologies [25].

Multiple additional studies highlight the crucial role of RWD and RWE in the HTA process. RWD significantly influences HTA submissions, particularly in Latin America, where Argentina and Brazil lead the way, and a rising trend is observed in Chile and Colombia [26]. In Europe and Canada, RWD contributes to HTA submissions for anticancer medications, providing comparative arms in Germany and supplementary evidence in Sweden and Canada [27]. Various European HTA organizations prioritize diverse clinical evidence, emphasizing the importance of RWD, especially in the initial reimbursement discussions, particularly for rare diseases. AIFA, ZIN, and HAS express a preference for RWD in conditional reimbursement schemes, acknowledging its positive impact on decision-making and effectiveness assessments [28]. European HTA representatives generally embrace registry data, with positive feedback on observational studies [29]. England shows a significant increase in RWD utilization in HTA submissions, with NICE leading in acceptance, and pragmatic trials and primary care databases recognized as valid sources [30–32]. Another study underscores extensive RWD use in evaluating the effectiveness and safety of Direct Oral Anticoagulants (DOACs) compared to warfarin in real-world clinical settings [33].

On the flip side, the impact of external control arms (ECAs) on the decision-making processes of drugs within well-known HTA organizations remains unclear and is likely minimal [34]. While concerns persist regarding the general collection of RWD, it is anticipated that the enactment of Germany’s new GSAV law will enhance the thoroughness of RWD collection [35]. RWD sources generally furnish relevant health outcomes data for the HTA process, but noticeable gaps exist in economic and comparator data, particularly in studies related to hip and knee arthroplasty [36].

In the realm of cost-effectiveness analyses (CEA), RWE from registries and statistical databases, especially for utility, cost, resource use, and quality of life data, proves crucial [37, 38]. Numerous European HTA organizations prioritize evidence from RWD for pharmacoeconomic analyses, giving emphasis to local RWD for costs, resource use, epidemiology, and quality of life [28]. Positive contributions to drug approval are noted with cost-effectiveness data from local registries [39]. However, a MENA-based study indicates opposition to the positive acceptance of RWD within the HTA framework [40].

### Barriers, challenges, and difficulties encountered in incorporating RWD/RWE within HTA

The challenges and barriers in utilizing RWD and RWE within HTA are diverse and multifaceted, spanning from issues in trial design, data quality, and methodological challenges to barriers related to stakeholder acceptance, industry engagement, and global harmonization efforts.

Methodological challenges associated with RWD and RWE include selection bias within international context [34], lower data quality compared to RCTs in various countries including several European countries and Canada [20, 33, 36, 41]. Standardization issues as well as design and reporting issues of non-RCT studies were also key concerns in European countries [18, 23] and limited infrastructure for collecting RWD with data collection issues in Canada for decision-making pertaining to drug pricing and reimbursement in Canada as well as in South American countries [26, 41], and concerns about the representativeness of RWD data [20].

The obstacles in utilizing RWE encompass bias and confounding factors, incomplete data availability and data accessibility challenges [24, 33, 35], study design and analysis when integrating into HTA [31], the absence of consensus on methodologies, and a shortage of qualified researchers [26]. Issues related to RWE include lack of reliability and bias as shown by stakeholders in Canada [42]. Internationally, there’s a scarcity of RWD for advanced therapies [43] and local RWD transferability challenges arizing in MENA countries [40]. An extra challenge in Norway is the exclusion of oncology data from the Norwegian Prescription Database, hindering the reassessment potential of registries [17].

Conversely, in the United Kingdom, there is a notable concern regarding the integration of primary care RWD into CEA models to support clinical inputs within the country’s HTA [30]. Simultaneously, the impracticality of conducting indirect comparisons among observational studies has been identified as a significant challenge [32]. In addition, barriers to increasing the use of RWD were the lack of experts/staff to analyze these data in South American countries as well as within international setting [19, 26].

### Potential benefits, opportunities, and feasibility of utilizing RWD/RWE in the HTA process

The role of registries in offering comprehensive documentation of disease progression and real-world treatment patterns as well as important data for HTA are highlighted within Norway, and international setting [17, 43], and post-marketing process through observational studies including registries are increasingly vital access strategies for certain technologies within UK’s health system [32]. Modeling limitations within CEA can be addressed through better guidance on registries data utilization [39]. The investment and reinforcement of patient registries that derive local data and payer databases within MENA countries [40] or primary care databases within UK are among the potential opportunities to enhance utilization of RWD. On the other hand, initiatives and advancements to address challenges in RWD and non-RCTs for HTA within Europe and Canada include standardized data elements, analytical methods with bias management, and data exchange platforms [23, 42]. Standardization for reliable RWD was also a key advancement reported in other studies for Europe [33, 36].

In regards to the utilization of evidence from non-RCTs in assessing treatment effects within the HTA process, stakeholders from several European countries recommended the enhancement of non-RCTs quality by justifying, designing, and managing rigorously, the improvement of HTA processes, skills, guidelines, and support of research for high-quality data [18]. European HTA organizations could strengthen the RWD but harmonization and alignment incentives are the main contributing factors [28]. Furthermore, evaluating evidence from single-arm trials is challenging and the need for guidelines and best practices is emphasized and it seems that RWD boosts acceptance, especially in oncology [31].

Additional potential benefits and opportunities of using RWD/RWE in HTA include enhancing understanding, complementing trials, careful data selection and rigorous study designs as well as the need for additional guidance on study design and adherence to best practice guidelines and integration of RWD into HTA for oncology medicines [19, 27, 34, 37]. The need for balancing the use of local and international RWE without delaying assessments shown by studies focusing in South American and Central and Eastern European countries (CEEC) [26, 44]. For South America, improved RWD data recording reported as crucial advancement. Within CEEC, collaboration and stability are crucial for successful RWE transferability and implementation.

The complementary role of RWE to strengthen evidence as another opportunity was reported within several studies focusing in United Kingdom, Asian countries and several European countries [20, 24, 25] while can also serve practical for outcome-based contracting [21]. The collaboration and stakeholder engagement need for RWD utilization improvement is an important aspect [19, 20, 24, 25, 36, 41, 45]. Last, but not least, vital components include implementing effective governance for RWE, establishing comprehensive registries and repositories, and demonstrating commitment to pragmatic trials, ensuring the robustness and reliability of RWE [35], potential usage of RWD and RWE for innovative technologies lacking ample evidence [29].

## Discussion

In this study, we conducted a review to comprehensively evaluate the available evidence on RWD and RWE for HTA process. The findings presented reveal a complex landscape in the current use and acceptance of RWD and RWE in HTA across various organizations and countries and illustrate the diversity in attitudes and preferences toward RWD and RWE among HTA organizations across different countries and regions. Several key observations and trends emerge from the diverse set of studies conducted. The acceptance and utilization of RWD and RWE display significant variations among HTA organizations globally. Prevailing preferences for RCTs and systematic reviews are evident in multiple studies, emphasizing a traditional approach to evidence. However, these preferences shift when RCTs are unavailable, leading to considerations of RWD from observational studies. This highlights a pragmatic approach, acknowledging the limitations of RCT availability. Notable disparities surface in different regions, exemplified by the skepticism in Saudi Arabia regarding the reliability of RWE compared to RCT outcomes. This skepticism suggests a cautious approach to embracing RWE in certain contexts. Conversely, Austrian, French, and English HTA organizations recognize the value of observational studies, particularly in situations where RCTs are impractical. This indicates a more open stance towards diverse forms of evidence. The influence of RWD on HTA submissions is pronounced, especially in Latin America, where Argentina and Brazil lead in adopting RWE and RWD significantly influences HTA submissions. This trend suggests a growing acknowledgment of the relevance and impact of RWD in decision-making processes. Similarly, in Europe and Canada, RWD contributes significantly to HTA submissions, providing additional evidence for medications, particularly in the context of anticancer treatments as presented within results. European HTA representatives generally embrace registry data, with positive feedback on observational studies while England shows a significant increase in RWD utilization in HTA submissions, with NICE leading in acceptance. Also, based on one study, it seems that Asian countries show a widespread positive inclination toward embracing and utilizing RWD and RWE by HTAs for assessing clinical effectiveness and reimbursing technologies and another study focusing in MENA indicates resistance to accepting RWD in HTA framework.

In contrast, this review concluded important findings in regards to the barriers and issues arize with the use and acceptance of RWD and RWE for HTA. These findings highlight the challenges faced in leveraging real-world data for informed decision-making in healthcare. One of the prominent barriers identified is the limited availability and transferability of local RWD. This limitation poses a challenge in accessing comprehensive and relevant data sources, particularly in specific regions or healthcare contexts. The lack of local data hinders the ability to generate evidence that is tailored to the specific needs and characteristics of the population under assessment. Accessing high-quality data is crucial for reliable and credible evidence generation. However, several studies revealed difficulties in accessing reliable and high-quality RWD. The challenges can arise from issues such as data privacy and confidentiality concerns, limited data standardization, and variations in data collection and reporting practices. These barriers undermine the reliability and credibility of the evidence derived from real-world data sources. Methodological challenges were also identified as a significant barrier to utilizing real-world data and evidence in HTA. Studies pointed out challenges in study design, analysis, and reporting when relying on non-randomized clinical evidence. Addressing these methodological challenges is crucial to ensure the validity and robustness of findings derived from real-world data sources. Insufficient expertise among stakeholders in utilizing RWE emerged as a common barrier. This lack of expertise can hinder the effective use and interpretation of RWD. Stakeholders, including policymakers, payers, and clinicians, need to possess the necessary skills and knowledge to critically evaluate and utilize RWE in decision-making processes. Fragmentation and lack of collaboration among stakeholders were found to hinder the utilization of real-world data. The absence of harmonized approaches, data sources, methodologies, and decision-making processes limit the consistent and efficient use of RWE. Enhancing collaboration and promoting standardization among stakeholders are essential for maximizing the potential of RWD in HTA. Data quality and reliability were highlighted as significant concerns. Studies identified issues related to low data quality, confounding biases, incomplete data, and challenges in data protection and confidentiality. These limitations can undermine the validity and generalizability of findings derived from RWD sources. Overall, the studies underscore the need to address these barriers and challenges to effectively utilize RWD and RWE in HTA. Improving data availability, ensuring data quality and standardization, addressing methodological challenges, promoting collaboration, and enhancing expertise among stakeholders are key considerations for advancing the use and acceptance of RWD in healthcare decision-making processes. The results of this review concerning to opportunities related to the RWD inclusion in HTA are in line with the literature and particularly with published manuscript of Crane G, et al. (2022) [46] whom results were similar and highlighted the importance of recommending approaches and initiatives for improving RWE utilization in healthcare decision-making in East Asia and beyond and Encouraging large-scale collaborations among government agencies, hospitals, research organizations, patient groups, and the pharmaceutical industry to ensure access to robust real-world data and alignment on addressing evidence needs.

The systematic review methodology employed in this study offers several strengths, enhancing the reliability and credibility of our findings. One of the key strengths of a systematic review is its comprehensive and rigorous approach. By adhering to a predefined and transparent methodology, we ensured that all relevant studies on the research question were identified, appraised, and synthesized. This minimizes bias and increases the validity of our results. An additional notable strength inherent to the systematic review methodology is its innate capacity to mitigate selection bias. This was achieved through the meticulous application of explicit inclusion and exclusion criteria, thereby effectively diminishing the prospect of selectively favoring studies that align with a particular perspective. Such an approach significantly bolsters the objectivity and neutrality of our review. Moreover, our systematic review facilitated the amalgamation of a diverse body of evidence. We thoughtfully incorporated studies employing a spectrum of methodologies, spanning various populations and settings. This comprehensive and inclusive approach underscores the robustness of our findings, fostering a more holistic perspective on the research question at hand. Despite these strengths, it is important to acknowledge the limitations of our systematic review. Firstly, although we aimed to conduct a comprehensive search, it is possible that some relevant studies may have been inadvertently missed, mainly due to the strict inclusion criteria of English-written manuscripts. This linguistic restriction may introduce a potential source of bias, as relevant studies or data published in languages other than English were not incorporated into our analysis, which could impact the comprehensiveness and generalizability of our findings. While conscientiously implementing strategies to mitigate bias within this systematic review, it is imperative to acknowledge that the specter of publication bias persists as a potential limitation. Despite our diligence in data collection and analysis, it is challenging to wholly obviate this concern, as it hinges upon the selective dissemination of research findings, rendering an absolute negation of its influence unattainable. Furthermore, while we have made every effort to conduct a comprehensive review, we recognize certain limitations that may impact the generalizability of our findings. The search strategy, while aiming for breadth and inclusivity, may exhibit some imbalance due to specific limitations in the selection of keywords. This imbalance could influence the identification and inclusion of relevant studies, potentially leading to an underrepresentation of certain perspectives. An additional limitation pertains to the specificity of the keywords used in our search strategy, which may have inadvertently led to the exclusion of relevant articles, particularly from the United States. We recognize that certain terms, such as “economic assessment of pharmaceutical” [47] may not have been adequately accounted for. In particular, the dataset under consideration primarily encompasses experiences from European Union (EU) countries, with limited representation from the United States. It is crucial to acknowledge that the selected studies included only a few instances reflecting the U.S. context. The scarcity of information from the US poses a challenge to the generalizability of our findings. This limitation is noteworthy as the United States, with its unique healthcare landscape and regulatory framework, plays a significant role in the global utilization of real-world evidence (RWE) for regulatory and reimbursement decisions. The dearth of comprehensive representation from the U.S. may restrict the broader applicability of our study outcomes.

The incorporation of RWE within submissions for HTAs has experienced a noticeable surge in recent times worldwide. In particular, based on an IQVIA analysis of 16,515 HTA reports from 83 HTA bodies in 33 countries, the percentage of records integrating RWE within submissions has increased significantly, from a mere 6% in 2011 to 39% in 2021. This indicates a substantial upward trend in the integration of RWE within submissions for HTA reports while several organizations publishing guidelines on how RWD can be used for HTA [48]. The notable increase, from 6% in 2011 to 39% in 2021, suggests a growing recognition and utilization of RWE as a valuable component in informing HTA processes across various countries and health organizations. The dynamic landscape revealed in this study emphasizes the evolving utilization of RWD and RWE within HTA, offering valuable insights for policymakers, healthcare professionals, and stakeholders grappling with the challenges and opportunities in today’s HTA assessments. By elucidating the current landscape of RWD/RWE acceptance and challenges across various HTA organizations and countries, these findings contribute valuable insights that can guide policy-makers, healthcare professionals, and stakeholders. This study outlines a spectrum of challenges associated with RWD/RWE, from methodological issues to stakeholder acceptance and infrastructure limitations with up-to-date data. By delineating these barriers, your research serves as a foundation for developing targeted interventions, guidelines, and capacity-building initiatives to enhance the integration of RWE into HTA processes. The study’s exploration of potential benefits, opportunities, and feasibility of RWD/RWE usage in HTA provides a forward-looking perspective. This can guide future research endeavors, policy developments, and collaborative efforts aimed at optimizing RWD/RWE contributions to HTA assessments. It is essential to underscore that the efficacy of systematic reviews is intrinsically tied to the caliber and lucidity of the underlying studies. Regrettably, any deficiencies or methodological shortcomings present within the primary studies inherently permeate our review, thereby potentially undermining the veracity of our collective findings. It is imperative to note that, by and large, the studies incorporated into our review demonstrated commendable quality, although a minority exhibited a moderate level of quality, signaling the importance of interpreting our findings within this context.

## Data Availability

The original contributions presented in the study are included in the article/Supplementary Material, further inquiries can be directed to the corresponding author.
