# Supplementary material for: Real-world data: a comprehensive literature review on the barriers, challenges, and opportunities associated with their inclusion in the health technology assessment process
Source: J Pharm Pharm Sci. 2024 Feb 28;27:12302. doi: 10.3389/jpps.2024.12302 (PMC10932954; doi:10.3389/jpps.2024.12302)
Supplement: Supplementary file 1 [file DataSheet2.pdf]

Abbreviations List

RWD: Real-World Data

RWE: Real-World Evidence

HTA: Health Technology Assessment

PRISMA: Preferred Reporting Items for Systematic Reviews and Meta-Analyses

IDEAS-RePEc: Internet Database of Economics Available at the Research Division of the Federal Reserve Bank of St. Louis

RCTs: Randomized Controlled Trials

REAs: Relative Effectiveness Assessments

ATMPs: Advanced Therapy Medicinal Products

COVID-19: Coronavirus Disease 2019

FDA: U.S. Food and Drug Administration

ICER: Institute for Clinical and Economic Review

CASP: Critical Appraisal Skills Programme

JBI: Joanna Briggs Institute

ECAs: External Control Arms

GSAV: Gesetz für mehr Sicherheit in der Arzneimittelversorgung (German Medicines Supply Act)

NICE: National Institute for Health and Care Excellence

MENA: Middle East and North Africa

CEEC: Central and Eastern European Countries

AIFA: Agenzia Italiana del Farmaco (Italian Medicines Agency)

ZIN: Zorginstituut Nederland (National Health Care Institute, Netherlands)

HAS: Haute Autorité de Santé (French National Authority for Health)

NESTcc: National Evaluation System for health Technology Coordinating Center

CDRH: Center for Devices and Radiological Health

ICER: Incremental Cost-Effectiveness Ratio

EMA: European Medicines Agency

IMI: Innovative Medicines Initiative

HRQL: Health-Related Quality of Life

CADTH: Canadian Agency for Drugs and Technologies in Health

PBAC: Pharmaceutical Benefits Advisory Committee (Australia)

AMNOG: Arzneimittelmarkt-Neuordnungsgesetz (German Pharmaceutical Market Reorganisation Act)

EU: European Union

SMC: Scottish Medicines Consortium

IQWiG: Institut für Qualität und Wirtschaftlichkeit im Gesundheitswesen (Institute for Quality and Efficiency in Healthcare, Germany)

TAVI: transcatheter aortic valve implantation

TMVR: transcatheter mitral valve repair

EC: External Comparator

FDA: Food and Drug Administration

HER: Electronic Health Record

CEA: Cost-Effectiveness Analysis

REA: Relative Effectiveness Assessment

ERG: Evidence Review Group

DOACs: Direct Oral Anticoagulants
