# Supplementary material for: Real-world data: a comprehensive literature review on the barriers, challenges, and opportunities associated with their inclusion in the health technology assessment process
Source: J Pharm Pharm Sci. 2024 Feb 28;27:12302. doi: 10.3389/jpps.2024.12302 (PMC10932954; doi:10.3389/jpps.2024.12302)
Supplement: Supplementary file 2 [file Table1.docx]

**Table 1. Characteristics of included studies**

| **Author/year** | **Country** | **Type of study** | **Objectives** | **Type of health technology** | **Population/therapeutic category** | **Subcategory/type RWD** |
| --- | --- | --- | --- | --- | --- | --- |
| Hagen G, et al (2021) | Norway | Scoping review | The Mapping of the Norwegian HTA System, including its Central Components and Partners, with an Emphasis on Different Sources of Evidence, such as Norwegian Patient Registries and the Recommendation for Using Observational Study Data at the Norwegian and International Level.  This study focuses on mapping the Norwegian HTA system, examining its central components and partners. | Pharmaceuticals, medical devices, procedures | General population/all therapeutic categories | Patient registry |
| Gonçalves E, (2020) | Internationally | Editorial | To present a comprehensive analysis of the current status of the evaluation of advanced therapy medicinal products and the integration of ethical evaluation into the HTA process. | Advanced Medicinal Products | Rare population/Rare diseases | RWD - General |
| Fasseeh A,  et al. (2020) | Middle East and North Africa | Primary research with questionnaires (qualitative) | To assess, through questionnaires to HTA experts, the current and future state (for the next 10 years) of HTA implementation in the Middle East and North Africa region, focusing on regional similarities rather than differences. | All health technologies | General population/all therapeutic categories | Patient registers and payer databases |
| Leahy T P,  et al. (2020) | United Kingdom | Systematic review | To investigate the use and acceptability of evidence derived from primary care databases, a key source of RWE in the UK, in NICE health technology assessments and to provide recommendations on their use in future submissions. | All health technologies | General population/all therapeutic categories | Primary care databases |
| Fuchs S, et al (2016) | Europe | Systematic review | To review and compare the existing activities of HTA for MD among European PTI (Payers, Technology Assessors, and Industry) organizations. The specific objectives are twofold: (i) to identify the institutions involved in MD-HTA across Europe, and (ii) to examine their structural, procedural, and methodological characteristics, with a specific focus on the assessment of medical devices. | Medical devices | General population/all therapeutic categories | Observational studies |
| Dai WF, et al, (2021) | Canada | Qualitative study using simulation | To conduct a simulation-based evaluation of a proposed re-evaluation process for cancer medicines in Canada. The proposed process was developed by the CanREValue stakeholder collaboration with the objective of improving the re-evaluation process. The stakeholders involved in this collaboration included health professionals, patient representatives, CADTH specialist staff, and other representatives from Canadian regulatory authorities. To evaluate the proposed process, a case study was conducted using real-world data on bevacizumab for metastatic colorectal cancer.  Through this simulation-based evaluation, the study aimed to assess the effectiveness and feasibility of the proposed re-evaluation process and its potential impact on decision-making regarding cancer medicines in Canada. | Medicinal products | Oncology patients/oncology | RWD - General |
| Patel D, et al, (2021) | Internationally | Review (Retrospective study) | To assess the current practices and challenges related to the use of external comparators in HTA submissions based on single-leg test data.  The assessment involved a retrospective review of 433 submissions from 100 HTA organizations across 40 countries. The review analyzed associated data collected between 2011 and 2019. The study seeks to provide insights into the utilization of external comparators in HTA submissions and highlight any challenges encountered during this process. | All health technologies | General population/all therapeutic categories | RWD: registers, databases, chart review and non-prescriptive study design |
| Tolley, K. (2010) | United Kingdom | Commentary | The primary aim of this article is to furnish a comprehensive overview of the challenges intertwined with the United Kingdom's Health Technology Assessment (HTA) system. This is achieved through a retrospective analysis of an HTA database, with a specific focus on scrutinizing the clinical evidence base in the assessment of novel pharmaceutical technologies within the UK context. The analysis focuses on issues related to clinical evidence requirements, cost-effectiveness evaluations, and the role of stakeholders in decision-making processes. By examining the clinical evidence base and identifying challenges within the UK's HTA system, the article aims to contribute to a better understanding of the evaluation process for new pharmaceutical technologies in the UK. | All health technologies | General population/all therapeutic categories | Non-randomized studies, observational studies |
| Kent, S. et al, (2021) | UK, Netherlands, Spain, Germany, Norway, Sweden, Austria, Italy | Pragmatic review and qualitative research | To provide recommendations on the appropriate use of evidence from non-randomized studies on treatment effects in the appraisal and technology identification (ATI) process.  To achieve this objective, pragmatic-type reviews and workshops were conducted involving 16 experts from appraisal and regulatory agencies across eight European countries. Through these reviews and workshops, the study aimed to gather insights and expertise to inform the development of recommendations regarding the utilization of evidence from non-randomized studies in assessing treatment effects within the HTA process. The input and perspectives of the experts involved are crucial in ensuring the relevance and applicability of the recommendations within the context of HTA evaluations. | All health technologies | General population/all therapeutic categories | Non-randomized studies |
| Jaksa A, et al, (2022) | Internationally | Literature review | To evaluate the critiques of regulatory agencies and HTA organizations regarding the use of external control arms (ECAs) with real-world data. The objective is to highlight the need for developing recommendations for the design and production of ECAs in the future. A review was conducted on the FDA website, analyzing drug and biologics approvals from 2018 to 2021. By examining the challenges and critiques faced by regulatory agencies and HTA organizations in utilizing ECAs with real-world data, the study seeks to contribute to the development of improved guidelines and practices for their use in future research. | Medicinal products | Oncology patients/oncology | External control arms designed with RWD (general) |
| Justo N, et al, (2019) | South America (Argentina, Brazil, Colombia, Chile) | Systematic review and primary qualitative research | To investigate and identify the sources, characteristics, and uses of RWD in Argentina, Brazil, Colombia, and Chile. The investigation also explores the implications of these findings for future legislation and the management of RWD. Additionally, workshop discussions were conducted with stakeholders to validate and verify the results obtained. By examining the sources and characteristics of RWD in these countries and considering stakeholder perspectives, the study provides valuable insights for the development of effective legislation and strategies for managing RWD in the future. | All health technologies | General population/all therapeutic categories | National health information systems, clinical registries (disease/condition specific), electronic health record systems, |
| Kamusheva M, et al, (2022) | Central  and Eastern Europe | Scoping review and qualitative research | To identify the main barriers to the implementation of evidence-derived RWE for the purposes of health technology assessment in Central and Eastern European countries. This is a mixed methods study using a literature review, internal discussions and a webinar with stakeholders from Central and Eastern European countries to identify barriers to the use of RWE in healthcare. | All health technologies | General population/all therapeutic categories | RWD - General |
| Timbie JW,  et al, (2021) | Internationally | Qualitative thematic  analysis research | To describe the current utilization of RWE for medical devices and assess the challenges faced by manufacturers regarding the generation and utilization of RWE for regulatory and reimbursement decisions. The investigation involved interviews with specific stakeholder categories, and a thematic analysis was conducted to identify key findings. The study provides insights into the existing use of RWE, highlights the obstacles encountered by manufacturers, and identifies opportunities for further utilization of RWE in the context of medical devices. By analyzing stakeholder perspectives, the study contributes to a better understanding of RWE's role and potential in regulatory and reimbursement decision-making processes. | Medical technologies | General population/all therapeutic categories | RWD and RWE  - General |
| Bullement A, et al, (2020) | England | Systematic review | The investigation of the utilization of RWE in informing STAs of cancer drugs conducted by the NICE. A review was conducted, spanning the period from April 2011 to October 2018.  The objective of the study is to examine how RWE has been incorporated into the assessment process for cancer drugs by NICE. By analyzing the use of RWE during this time frame, the study aims to provide insights into the role and impact of RWE in informing STAs of cancer drugs conducted by NICE. | Medicinal products | Oncology patients/oncology | RWD and RWE  - General |
| Al-Omar HA, et al, (2021) | Saudi Arabia | Scoping review and primary qualitative research | Τo explore the perspectives of a multi-stakeholder group comprising local experts regarding potential value-adding elements that could be applicable to HTA processes and methods for pharmaceutical products in Saudi Arabia.  To achieve this objective, a review and workshop were conducted using a pooling system for data collection. The study seeks to gather insights from a diverse range of stakeholders to identify and assess elements that can enhance the effectiveness and relevance of HTA processes and methods specifically for pharmaceutical products in the Saudi Arabian context. By engaging stakeholders and utilizing a pooling system, the study aims to provide valuable input for the improvement of HTA practices in Saudi Arabia. | Medicinal products | General population/all therapeutic categories | RWD and RWE - General |
| Makady A, et al, (2018) | England, Scotland, Netherlands, France, Germany | Literature review (retrospective study) | This study examines the use of RWD in the HTA process. Specifically, it focuses on the inclusion of RWD in the REAs and CEAs of melanoma drugs by five HTA organizations in Europe.  The study investigates the differences and similarities in the utilization of RWD, including the types of RWD employed, sources of data, methodological approaches, and the challenges faced. It also explores the potential benefits and limitations associated with incorporating RWD in HTA. By analyzing these aspects, the study contributes to a better understanding of the role and impact of RWD in HTA and identifies areas for improvement in the utilization of RWD for melanoma drug assessments. | Medicinal products | Oncology patients/oncology | RWD and RWE - General |
| Deverka PA, et al, (2020) | US | Literature review | This study endeavor sought to elucidate the contemporary panorama concerning the utilization of real-world evidence (RWE) by payers in the context of shaping their coverage determinations, while also exploring prospective remedies aimed at surmounting associated impediments. | Next generation sequencing (NGS) test | General population/all therapeutic categories | RWD and RWE - General |
| Lou J, et al, (2020) | Asia (Bhutan, China, China, India, Indonesia, Japan, Malaysia,  Philippines, Singapore, Singapore, South Korea, Taiwan and Thailand) | Primary qualitative research | The primary objective of this paper was to address the growing interest in using RWD and RWE for HTA in Asia, by conducting several activities to gather personal and health system level experiences of using RWD/RWE to inform HTA for reimbursement decisions in eleven health systems in Asia. It highlights the need for a conceptual framework to standardize the collection, analysis, and utilization of RWD and RWE in the region. It aims to inform HTA processes and proposes the establishment of an international collaboration called the REAL World Data In ASia for HEalth Technology Assessment in Reimbursement (REALISE) working group to provide guidance on using RWD and RWE in decision-making for healthcare technologies in Asia.  The survey was conducted in three stages: a distance meeting (online), a face-to-face meeting and a videoconference. | All health technologies | General population/all therapeutic categories | RWD and RWE - General |
| Facey KM, et al, (2020) | European Union countries | Primary qualitative research | The study investigates highly innovative technologies that pose a challenge for payers and HTA organizations, as they have to make decisions based on limited evidence and significant uncertainties. The objective is to explore potential actions that stakeholders can take to enhance the utilization of RWD in this specific environment, focusing on the decision-making process from the perspective of payers and HTA organizations. A mixed methods approach was employed, involving an examination of recent policy proposals regarding RWD use in payer/HTA decisions. Stakeholder engagement took place through workshops, teleconferences, and email consultations. | All health technologies | General population/all therapeutic categories | RWD and RWE - General |
| Bowrin K, et al, (2019) | Internationally | Systematic review | The study aims to examine the constraints associated with utilizing RWE in decision analysis, particularly in modeling, while also identifying existing recommendations for RWD-based modeling. It explores various aspects, including the current limitations of real-world studies, the application of real-world evidence in the context of RWE, the presence or absence of guidelines, and provides recommendations based on these findings. The study seeks to shed light on the challenges and provide guidance for the effective utilization of real-world data in decision analysis and modeling processes. | All health technologies | General population/all therapeutic categories | RWD and RWE - General |
| Brogaard N, et al, (2021) | England, Germany, France, Canada, Denmark, Sweden and Scotland | Literature review | An analysis was conducted on the HTA agency reviews and reimbursement decisions for entrectinib and larotrectinib in multiple countries, including England, France, Germany, Canada, Denmark, Sweden, and Scotland. The objective was to examine and compare the assessments and reimbursement outcomes of these two medications across the selected countries. By analyzing the HTA agency reviews and reimbursement decisions, the study aimed to gain insights into the similarities and differences in the evaluation and acceptance of entrectinib and larotrectinib among these countries. | Medicinal products | Oncology patients/oncology | RWD and RWE - General |
| Hogervorst Milou A, et al, (2022) | European countries | Primary qualitative research | The study aimed to assess the challenges associated with the acceptance of RWD in the context of new and complex health technologies. A survey was conducted by distributing questionnaires to representatives of HTA organizations. The objective was to gather insights into the specific obstacles that make the acceptance of RWD more likely for HTA evaluations. By examining the responses and feedback from HTA organization representatives, the study sought to identify the key challenges and barriers that hinder the wider utilization of RWD in the assessment of new health technologies.  . | All health technologies | General population/all therapeutic categories | RWD and RWE - General |
| Sievers H, et al, (2021) | Germany, England, Belgium, Sweden, Netherlands | Primary qualitative research | The study aimed to assess stakeholder perceptions regarding the challenges and value of evidence derived from post-marketing RWE. It also examined the differences in requirements for RWD collection between regulatory and HTA organizations in Germany under the Regulation for Greater Safety in Medicines Supply (GSAV). Additionally, the study explored future coordination opportunities to establish a complementary framework for post-marketing requirements related to RWE. The research methodology involved conducting semi-structured interviews with stakeholders, which were conducted via conference calls. The interviews provided valuable insights into the perspectives of various stakeholders, contributing to a comprehensive understanding of the challenges, opportunities, and potential coordination efforts related to the utilization of post-marketing RWE. | All health technologies | General population/all therapeutic categories | RWD and RWE - General |
| Hampson G, et al, (2018) | US | Literature review and primary qualitative research | To explore the current utilization of RWE in the US healthcare system, summarize key concerns raised in this field, and identify opportunities that could arise from improved use of RWE for reimbursement decisions. The research involved conducting a review of literature and data collection on the challenges and opportunities associated with RWE. Additionally, telephone interviews were conducted with nine RWE experts from the pharmaceutical industry, payers, and academia. These interviews provided valuable insights into the current landscape of RWE utilization, the concerns surrounding its use, and the potential opportunities that can be realized through its improved application in reimbursement decisions. | All health technologies | General population/all therapeutic categories | RWD and RWE - General |
| George E, (2016) | UK | Perspective/ opinion/commentary | The primary objective of this paper was to elucidate how the NICE incorporates evidence from sources beyond RCTs in its decision-making process, by identifying cases, and emphasizing NICE's pivotal role in guiding healthcare practices and resource allocation in England. | All health technologies | General population/all therapeutic categories | RWD - General |
| Makady E, et al, (2017) | England, Sweden, Germany, Italy, Netherlands, France | Literature review and primary qualitative research | The objective of this study was to investigate the policies implemented by six (6) HTA agencies in Europe concerning the utilization of RWD in the assessment of medicines through the process of REA. Specifically, the article examines the policies of these organizations regarding the acceptance or requirement of RWD, as well as their policies concerning the assessment of RWD in three distinct contexts: IRDs, PEAs, and CRS.  Additionally, the study involves conducting semi-structured interviews with representatives from the six (6) HTA agencies. | All health technologies | General population/all therapeutic categories | RWD - General |
| Husereau, D, et al, (2019) | Canada | Primary qualitative research | To obtain stakeholders' perspectives on the utilization of RWD in decision-making pertaining to drug pricing and reimbursement in Canada, and to identify the obstacles and enablers to the application of RWD in this context, this study aims to conduct qualitative research. Additionally, the study seeks to extract valuable insights that could be beneficial to other countries contemplating the use of RWD for similar objectives. The qualitative study will involve conducting semi-structured interviews and organizing focus group discussions with a diverse range of stakeholders, including payers, researchers, patient groups, and industry representatives. | All health technologies | General population/all therapeutic categories | RWD - General |
| Pongiglione B, et al, (2021) | Europe (15 countries) | Systematic review | The aim of this study was to investigate the availability and quality of real-world data concerning the documentation production for the Life Cycle Information Systems (LIS) of medical devices in Europe. The study primarily focuses on mapping and conducting a critical evaluation of existing real-world data sources in Europe that are relevant to the process of medical device-associated ATHENA (Assessment of Therapeutic Effectiveness by National Authorities).  In particular, the researchers concentrate on three specific cases: hip and knee arthroplasty, TAVI, and TMVR, as well as da Vinci robotic surgery procedures. | Medical technologies | General population/all therapeutic categories | 1. Administrative data  2. Registry data  3. Other data from observational studies  4. Other data |
| Ciminata, G, (2019) | Scotland | Diploma thesis including (i) an economic evaluation case study, (ii) a systematic review | The aim of this research was to investigate the potential advantages and obstacles associated with utilizing RWE to support the ATY. Specifically, the study focuses on exploring the viability of employing RWD in the decision-making process of HTA in Scotland, with a specific focus on cases involving anticoagulant medication for patients with atrial fibrillation.  Furthermore, the study aims to examine the perspectives of health technology evaluators regarding the utilization of real-world data in evaluating anticoagulant drugs for atrial fibrillation. The research also provides suggestions for enhancing the application of real-world data in health technology evaluation. | All health technologies | Cardiac patients/Cardiological diseases | RWD and RWE - General |

HTA: Health Technology Assessment, RWE: Real-World Evidence, NICE: National Institute for Health and Care Excellence, MD: Medical Devices, CADTH: Canadian Agency for Drugs and Technologies in Health, FDA: Food and Drug Administration, RWD: Real-World Data, STAs: Single Technology Assessments REAs: Relative Effectiveness Assessments, CEAs: Cost-Effectiveness Assessments, GSAV: Regulation for Greater Safety in Medicines Supply, RCTs: Randomized-Controlled Trials, CRS: Conditional Reimbursement Schemes, IRDs: Initial Reimbursement Discussions, PEAs: Pharmacoeconomic Assessments ATHENA: Assessment of Therapeutic Effectiveness by National Authorities, ATY: Assessment of Therapeutic Yield, TAVI: transcatheter aortic valve implantation, TMVR: mitral valve repair

Note: RWD and RWE – General encapsulates a comprehensive scope encompassing all varieties and subcategories of RWD and RWE.
