# Supplementary material for: Real-world data: a comprehensive literature review on the barriers, challenges, and opportunities associated with their inclusion in the health technology assessment process
Source: J Pharm Pharm Sci. 2024 Feb 28;27:12302. doi: 10.3389/jpps.2024.12302 (PMC10932954; doi:10.3389/jpps.2024.12302)
Supplement: Supplementary file 4 [file Table2.docx]

**Table 2. Overview of Current Utilization and Acceptance of RWD and RWE in HTA**

| **Author/year** | **Current use and acceptance** |
| --- | --- |
| Hagen G, et al (2021) | Today, multiple HTA organizations have released guidelines regarding the gathering and utilization of RWD. Within the scope of HTA, the primary recommended sources of clinical effectiveness data for evaluating the economic value of a health technology are systematic reviews and randomized clinical trials. When trials are not available, data from observational studies are utilized. These data sources inform various aspects of HTA, from economic evaluations to treatment patterns. RWD and RWE's expanding roles in HTA are anticipated, underpinning robust health policy decisions. The preference for RCTs as the primary source of clinical evidence is undergoing a global shift, especially in situations where challenges arise in trial design (particularly in the case of rare diseases), blinding (involving medical devices and procedures), and ethical considerations. Registries play a crucial role in crafting robust Health Technology Assessment (HTA) reports, and the utilization of registry data in this context is expected to grow in the coming years. |
| Fasseeh A, et al. (2020) | Local data utilization in the HTA process is not obligatory for most participants (84%), indicating limited acceptance or requirement in the current HTA framework. |
| Leahy T P, et al. (2020) | Among the 52 HTA submissions examined, databases were commonly used for describing disease epidemiology, treatment practices, and clinical inputs for cost-effectiveness models. |
| Fuchs S, et al (2016) | All organizations generally express a strong preference for direct evidence obtained from RCTs, but they also acknowledge and may recommend considering evidence from various study designs under specific circumstances. The Austrian Ludwig Boltzmann Institute for HTA emphasizes that, when investigating specific research hypotheses related to biomarker tests, the only practical and ethically acceptable evidence should be derived from observational studies. Different levels of hierarchy can be applied in evaluating such evidence. HAS acknowledges that comparative non-randomized observational studies may be acceptable in cases where conducting a randomized controlled trial is not feasible. However, such studies should be considered as exceptions.  Furthermore, NICE states that, when assessing the accuracy of a diagnostic test, it takes into account prospective cohort studies, synchronous studies, and retrospective case-control studies. On the other hand, the Medical Technology Assessment Programme evaluates primary and secondary research, including modeling studies and post-analysis, to form its assessments. |
| Patel D, et al, (2021) | The use of RWD/RWE in single-arm submissions to HTA is relatively low (5%), indicating a lack of widespread adoption.  The largest proportion of submissions were for oncology and haematology-oncology diseases (65% of ΗΤΑ submissions). Approximately half (52%) of the deposits included a dataset based on external comparators, of which 20% of these deposits (87 deposits) contained exclusively RWD with clear reference to the type of RWD source with undefined RWD studies dominating followed by a combination of RWD (37) and prior trials (35), registries (24), databases (23) and chart reviews (3).  Of note, between 2015-2019 there is a significant increase in the use of external comparators derived from RWD in contrast to the decrease recorded in the use of ECs from previous trials.  HTA organizations vary in their acceptance of submissions with external comparators. NICE has the highest acceptance rate for submissions with RWD-derived external comparators,followed by HAS, G-BA, CADTH, PBAC. In addition, the same organisations also showed a significantly high acceptance rate for submissions with ECS based on a combination of RWD and previous clinical trials. |
| Tolley, K. (2010) | Prior to 2010, a limited number of positive evaluations, often accompanied by conditions, were identified that were based on observational study data from NICE or evaluations conducted by academics for NICE. In recent years, the NICE agency has shown support for the inclusion of pragmatic-type trials, recognizing their potential to generate evidence from real-world technology usage. |
| Kent, S. et al, (2021) | Most HTA bodies state a strong preference for evidence on treatment outcomes from RCTs over non-randomised studies due to lower risk of impartiality. The quality of the non-randomised studies accepted so far by the HTA agencies for decision making varies |
| Jaksa A, et al, (2022) | Level of influence of external control arms (ECAs) in the decision-making process of HTA organizations, by drug:  1) Blinatumomab Ph- ALL - Low influence from NICE, G-BA, HAS, pCODR; High from PBAC  2) Abelumab - High influence from NICE and PBAC; Low from G-BA and HAS  3) Blinatumomab MRD + ALL - Low influence from NICE, G-BA, HAS, pCODR; High from PBAC  4) Erdafitinib - Not applicable for all HTA organizations  5) Endectinib - Low influence from NICE and pCODR; Not applicable for others.  6) Fam-trastuzumab deruxtecan-nxk - Low influence from NICE, HAS; Not applicable for G-BA, pCODR, PBAC  7) Idecabtagene vicleucel - Low influence from NICE, HAS; Not applicable for G-BA, pCODR, PBAC |
| Justo N, et al, (2019) | Argentina: RWD is utilized in HTA submissions in Argentina, and there is an anticipated significant increase in the use of RWD in financial assessments. Additionally, the pharmaceutical industry is funding cost-effectiveness studies based on RWD.  Brazil: RWD is regularly incorporated into HTA submissions to the national HTA agency (CONITEC) in Brazil. These submissions primarily involve epidemiological, resource utilization, and treatment data sourced from specific databases. Notably, between 2011 and 2014, 88.2% of the submissions included RWD.  Colombia: RWD is predominantly employed in economic evaluations, particularly in modeling exercises .  Chile: anticipates increased RWD use for HTA, balancing international and local data from registries concerning resource utilization and cost information. |
| Timbie JW, et al, (2021) | Payers prefer data from RCTs and have concerns about possible bias on the part of RWE. More generally, RWE has not yet served as the primary source to support a pre-market approval. |
| Bullement A, et al, (2020) | RWE integration is common in HTA evaluations (96%) to support the cost-effectiveness analysis within the HTA process.  In ten HTA evaluations, RWE was specifically included in response to requests or criticisms from the Evidence Review Group (ERG) or the Panel. It was notably for utility, cost, resource use, and effectiveness comparisons. |
| Al-Omar HA, et al, (2021) | Significant variations were observed, particularly in the mean scores for the acceptability and applicability of RWE in HTA with 11.1% expressing rejection of obligatory RWE inclusion for validating results from RCTs, and 13.9% opposing mandatory RCT data in the HTA process.. |
| Makady A, et al, (2018) | RWD predominantly originated from national registers and statistical databases.  In REAs, RWD was used in 54%, primarily assessing melanoma prevalence and incidence.  In CEAs, RWD contributed to 88%, extrapolating efficacy beyond RCT durations, estimating long-term effectiveness, cost data, resource use, and quality of life in specific cases. However, assessments for certain parameters in both REAs (33%) and CEAs (32%) lacked identification.  Among NICE, SMC, IQWiG, HAS, and ZIN, RWD utilization varied. All utilized RWD, with SMC making lesser use. Notably, RWD usage in both REAs and CEAs significantly increased from 2011 to 2016. However, only 2 out of 58 RWD submissions were linked to initial HTA submissions. |
| Deverka PA, et al, (2020) | RWE, acknowledged for its value, faced limitations in NGS coverage determinations. Payers leaned on clinical guidelines and RCTs, not RWE.Collaboration among stakeholders, awareness of data and methodological advancements, and the presentation of credible studies relevant to payer decision-making can contribute to an increased and more effective utilization of RWE by payers. |
| Lou J, et al, (2020) | In assessing RWD and RWE acceptance by HTA organizations, all 14 respondents favored their use for clinical effectiveness assessment. However, justifying RWD usage and acceptance still require attention.  In particular , when asked about the acceptance of data from real-world trials, 7 out of 14 respondents provided positive responses, indicating that acceptance is already in place. The remaining respondents mentioned that acceptance of real-world trial data is planned but not expected to happen soon. Survey results indicated positive attitudes towards RWD and RWE, though with nuances. |
| Bowrin K, et al, (2019) | According to one study included in Bowrin’s systematic review, all HTA organizations rely on RWE for documentation on costs, epidemiological data, and model transition probabilities.  RWE influenced decision-making, with 80% incorporation in UKsingle technology appraisal (STI) submissions. In 10% of these, RWE was used to inform clinical effectiveness and safety of the technology, while in a larger proportion it was used to report survival and resource use data.  RWE was shown to have a direct influence on decision-making, drug access and licensing depending on the HTA organisation - a prime example was the approval of a drug by the Pharmaceutical Benefits Advisory Committee of Canada, which was attributed to cost-effectiveness data derived from registries. |
| Brogaard N, et al, (2021) | RWD inclusion in HTA submissions (Germany, Sweden, Canada) faced methodological issues (Germany), aided evaluation (Sweden), and provided supplementary evidence (Canada). |
| Hogervorst Milou A, et al, (2022) | Survey respondents accepted RWD from patient registries (86%) and observational studies (55%). However, acceptance varied for case-control studies (13%) and cross-sectional studies (12%). Unpublished data and expert opinions received lower acceptance rates (25-28%). |
| Sievers H, et al, (2021) | According to five (5) respondents representing HTA organizations, pharmaceutical companies, and academia, RWE does not play a significant and scientifically decisive role in the evaluation of health technologies. Instead, the use of data from RCTs is preferred. Preference for RCT data persisted, especially for the German AMNOG system. Methodological challenges and infrastructure gaps were highlighted. |
| Hampson G, et al, (2018) | All interviewees highlighted methodological issues and the lack of robust infrastructure for collecting RWD as significant challenges. The implementation of Germany's GSAV law aimed to enhance RWD collection comprehensiveness, learning from other countries' patient registry experiences.However, Harmonization of documentation requirements between countries faced legal complexities.Nonetheless, there is a possibility of aligning data collection methodologies, which can facilitate better comparability and collaboration across countries. |
| George E, (2016) | Non-randomized clinical trials, observational studies, and pragmatic-type trials were normative in NICE HTAs, especially for devices.  RCTs may have limitations in terms of sample size, duration of follow-up, various comparators, crossover and statistical power. As a result, non-RCT clinical data are often used, especially for devices, interventions with difficult RCTs and conditions with poor prognosis. |
| Makady E, et al, (2017) | Initial Reimbursement Discussions: All six (6) HTA organizations accept all types of clinical evidence regarding REA, including RWD. HTA organizations require comprehensive documentation of RWD use (in cases where RCTs are not used for drug effectiveness or in cases of supplementary RWD use for subgroups). Comprehensive documentation and bias assessments were required, with impacts higher for rare diseases.  Pharmacoeconomic Evaluations: RWD was requested for Pharmacoeconomic Evaluations, recommending local RWD. Modeling required RCT data but could be supplemented with RWD.  Conditional Reimbursement Schemes: Conditional Reimbursement Schemes favored RWD, showing a positive impact on decision-making.Τhree HTA organizations (AIFA, ZIN, HAS) that have implemented such schemes reported a preference for these data. The Italian organization mentioned its participation in RWD collection and support for the applicant. Additionally, the positive impact of RWD on decision-making for conditional reimbursement was noted by all three organizations. |
| Pongiglione B, et al, (2021) | RWD sources with health outcomes data: In the mapping exercise, nearly all RWD sources had health outcomes data relevant to HTA. However, for hip and knee arthroplasty, four studies lacked or couldn't retrieve RWD. Conversely, TMVR, TAVI, and da Vinci robotic surgery procedures had HTA-related RWD from all sources except three German ones.  RWD sources with resource use data:  For hip and knee arthroplasty, nearly one-third of the registries and over half of the observation sources did not include economic data. Among the RWD sources analyzed, the most cited elements were length of hospital stay and type of procedure, while some references were made to hospitalization costs and procedure costs.  RWD sources with comparators data:  In the case of hip and knee arthroplasty, eighteen RWD sources did not present comparable data suitable for HTA. For TAVI/TMVR, the number was thirty-one sources, and for da Vinci procedures, it was twenty-six sources. |
| Ciminata, G, (2019) | RWDs demonstrated the effectiveness and safety of DOACs compared to warfarin in real-world clinical settings, providing robust evidence for their favorable profile. |

HTA: Health Technology Assessment, RWD: Real-World Data, SAT: Single-Arm Trials, HAS: French National Authority for Health (Haute Autorité de Santé), NICE: National Institute for Health and Care Excellence, G-BA: Gemeinsamer Bundesausschuss (Federal Joint Committee, Germany), CADTH: Canadian Agency for Drugs and Technologies in Health, PBAC: Pharmaceutical Benefits Advisory Committee (Australia), AMNOG: Arzneimittelmarkt-Neuordnungsgesetz (German Pharmaceutical Market Reorganisation Act), GSAV: Gesetz für mehr Sicherheit in der Arzneimittelversorgung (Law for Greater Security in Drug Supply, Germany), SMC: Scottish Medicines Consortium, IQWiG: Institut für Qualität und Wirtschaftlichkeit im Gesundheitswesen (Institute for Quality and Efficiency in Healthcare, Germany), ZIN: Zorginstituut Nederland (National Health Care Institute, Netherlands), AIFA: Agenzia Italiana del Farmaco (Italian Medicines Agency), TAVI: transcatheter aortic valve implantation, TMVR: transcatheter mitral valve repair, DOAC: Direct Oral Anticoagulation
