# Supplementary material for: Real-world data: a comprehensive literature review on the barriers, challenges, and opportunities associated with their inclusion in the health technology assessment process
Source: J Pharm Pharm Sci. 2024 Feb 28;27:12302. doi: 10.3389/jpps.2024.12302 (PMC10932954; doi:10.3389/jpps.2024.12302)
Supplement: Supplementary file 5 [file Table3.docx]

**Table 3. Barriers, challenges, and difficulties encountered in incorporating RWD/RWE within HTA**

| **Author/year** | **Barriers, challenges, and difficulties** |
| --- | --- |
| Hagen G, et al (2021) | The preference for RCTs as the primary source of clinical evidence is globally shifting, especially in challenging situations like rare diseases, blinding issues, and ethical considerations.. Challenges arise in reconciling limited oncology treatment data and ensuring their inclusion in analysis due to database limitations. |
| Gonçalves E, (2020) | For advanced therapies, clinical trials may lack long-term results. The absence or scarcity of RWD for advanced therapies necessitates reliance on outcomes modeling, indirect comparisons, and extrapolations, posing challenges in continuous data collection and reliability management.  .  Demonstrating the long-term effectiveness and comparative benefits of ATMPs requires continuous data collection and analysis. Managing data collection and ensuring its reliability can be a substantial challenge, including collection of RWD. |
| Fasseeh A, et al. (2020) | HTA organizations endorse leveraging comparative effectiveness data but acknowledge inherent limitations. Adaptation to locally relevant data becomes imperative, but challenges arise due to a scarcity of local RWD from patient registries and restricted access to payer databases. |
| Leahy T P, et al. (2020) | The committees predominantly raised comments and sought clarifications regarding the incorporation of documentation derived from primary care databases into cost-effectiveness models, particularly in relation to informing clinical inputs. Both the ERG (Evidence Review Group) and the committees expressed concerns when data from a published study were repurposed for the NICE submission. Analyses specifically conducted to inform the NICE submission were better received by both the ERGs and the review committees. |
| Fuchs S, et al (2016) | Standardizing RWD use across institutions faces barriers due to varying understanding and acceptance of different study designs. Limited guidelines for Medical Device (MD) assessment contribute to difficulties in creating a unified framework for RWD usage in HTA. |
| Dai WF, et al, (2021) | Challenges in RWE Utilization include:  A. Stakeholders deem RWE from individual institutions unreliable due to limited scope, lack of generalizabilityand potential bias  B. Ensuring representation and reliability in Canadian populations via adherence to accepted procedures.  C. Cost-Effectiveness Threshold Challenges in Canada: Establishing a cost-effectiveness threshold (Incremental Cost-Effectiveness Ratio or ICER) for re-evaluations in Canada poses challenges. |
| Patel D, et al, (2021) | Acceptance of RWD and RWE varies among HTA stakeholders, influenced by differing levels of maturity and familiarity.  Challenges arise in study design and analysis when incorporating RWD/RWE into HTA assessments. Ongoing research on statistical methods for external comparison suggests that there are methodological challenges in effectively utilizing RWD/RWE. Publication bias may be a factor, as some HTA bodies do not publish their evaluations, and the availability of information on documentation, including RWD/RWE, may be limited in certain cases. |
| Tolley, K. (2010) | UK HTA agencies have specific requirements for demonstrating clinical effectiveness and cost-effectiveness.  Although pragmatic-type trials aim to strike a balance between internal and external validity, they still present challenges. Challenges arise in utilizing indirect or mixed treatment comparisons in observational studies, impacting practicality. |
| Kent, S. et al, (2021) | Issues associated with utilizing non-randomized clinical evidence to assess treatment effects in HTA include:  A. Study Planning and Design: The primary challenges encountered during these studies revolve around the planning and design phase. This encompasses errors stemming from participant selection, data limitations, confounding factors, and the adequacy of the sample size.  B. Analysis: Analyzing non-randomized data presents several key problems. These include potential errors arising from participant selection biases, limitations in available data, confounding variables, and insufficient sample sizes. Addressing these challenges requires robust analytical methods. Unclear Reporting of Data,  C. Methods, and Results: Another critical issue is the lack of clarity in reporting the data, methods employed, and the resulting outcomes of non-randomized studies. This ambiguity hinders the assessment of the study's reliability and replicability.  D. Absence of Systematic Protocols: The absence of systematic protocols for designing non-randomized studies is identified as a significant concern. The lack of standardized protocols can lead to inconsistencies in study design, implementation, and reporting, posing challenges in evaluating and comparing the results across studies. |
| Jaksa A, et al, (2022) | The ΗΤΑ organizations have provided specific ratings for external control arms (ECAs) for various drugs, in terms of challenges and issues:  1) Blinatumomab - NICE: Unmeasured confounding, G-BA: Inconsistent standard of care over time, HAS: Unmeasured confounding, inconsistent outcome definitions, pCODR: Inconsistent standard of care over time, unmeasured confounding, selection bias, PBAC: Inconsistent pattern of care over time, unadjusted confounders, incorrect adjustment methods  2) Abelumab - NICE: Selection bias, G-BA: Selection bias, HAS: Inconsistent outcome definitions pCODR: Not specified, PBAC: Data loss/inadequacy  3) Blinatumomab MRD + ALL - NICE: Non-generalizability of ECAs in clinical practice,G-BA: Selection bias, HAS: Not specified, pCODR: Inconsistent pattern of care over time, unadjusted confounders, PBAC: Inconsistent pattern of care over time, non-generalizability of ECAs in clinical practice, unmeasured confounding, selection bias, loss/inadequate data  4) Erdafitinib - No rating provided by any HTA organization  5) Entrectinib - NICE: Not specified, G-BA: Non-generalizability of ECAs in clinical practice, unmeasurable confusion, HAS: Not specified, pCODR: Unmeasurable confounding, selection bias, PBAC: Not specified  6) Fam-trastuzumab deruxtecan-nxk - NICE: Not specified, G-BA: Not specified, HAS: Unmeasured confounding, pCODR: Not specified, PBAC: Not specified  7) Idecabtagene vicleucel - NICE: Not specified, G-BA: Not specified, HAS: Unmeasurable confusion, loss/inadequate data, pCODR: Not specified, PBAC: Not specified |
| Justo N, et al, (2019) | Quality, Consistency, and Collection Method Issues: There are issues and difficulties regarding the quality, consistency and method of collection of RWD, and there is a lack of expertise and training of staff. In addition, there is also a lack of confidence in observational research as good practices are often not followed and procedures are not transparent.  Expertise and capacity: There is a lack of qualified staff with the expertise to analyse the large volume of RWD available. |
| Kamusheva M,  et al, (2022) | Technical barriers:  A. Lack of expertise and capacity in HTA organizations for adequate RWE assessment.  B. Limited financial resources for RWE utilization and management.  C. Inadequate methods and practices for reporting RWD diligence.  Regulatory barriers:  D. Absence of unified, widely accepted, and implemented guidance documents for publishing and sharing RWE across all EU countries.  E. Lack of cooperation and standardized data integration practices for common RWE throughout Europe.  F. Requirements mandating the use of only local documentation in HTA.  G. Unclear and ambiguous requirements regarding the appropriate utilization and timing of RWE.  H. Insufficient local/national governance framework supporting the use of RWE.  I. Frequent changes in regulations related to RWE.  Clinical/scientific barriers:  J. Unique demographic, racial, ethnic, and genetic characteristics pose challenges for RWE analysis.  K. Variations in epidemiological data exist among different countries.  L. Differences in disease severity affect the transferability of RWE for specific patient groups.  M. Variations in medical practices hinder the applicability of RWE across regions.  N. Divergent predefined criteria for assessing the effectiveness of drugs complicate RWE evaluation.  O. Lack of transparency in the planning, execution, and reporting of RWD use.  P. Absence of established methodological standards for curating RWD.  Q. Uncertainty surrounding the results of RWE studies.  Other obstacles:  R. Uncertainty about the quality of RWE.  S. Limited confidence in RWE due to restricted access to RWD.  T. Variation in the impact and importance of RWE in decision-making processes across different CEE countries. |
| Timbie JW, et al, (2021) | The industry faces several challenges, which impede the effective use of RWE and particularly include:  A. Uncertain return on investment, causing hesitation in investing in RWE due to a lack of clarity on how the FDA assesses its value.  B. Difficulty in finding partners with large-scale and high-quality data, as researchers and customers often have limited access to relevant data. This can lead to delays in establishing new partnerships.  C. Insufficient staff with expertise in RWE to identify appropriate sources of RWD and conduct thorough analyses. This limitation hampers the exploration of RWE utilization.  D. Limited availability of unique device identifiers in RWD, particularly in electronic file systems and contracts, posing challenges for device-specific analysis.  E. Inadequate recording of longitudinal data with a short follow-up period, restricting the ability to capture comprehensive and meaningful information over an extended period of time.  F. Difficulties in linking data from different sources, hindering efforts to integrate and analyze data from multiple perspectives. |
| Bullement A,  et al, (2020) | Critique of RWE sources, which highlight potential biases when interpreting data collected outside of RCTs:  A. The comparability of real patient cohorts to those in clinical trials and  B. the relevance of RWE patient cohorts to current medical practice. |
| Al-Omar HA,  et al, (2021) | A. Lack of availability and quality of RWD: One of the prominent barriers identified in Saudi Arabia is the limited availability and inadequate quality of RWD. This hinders the effective utilization of such data in healthcare decision-making processes.  B. Concerns about data privacy and confidentiality: Workshop participants expressed apprehensions regarding data privacy and confidentiality. Ensuring the protection of patient information and maintaining confidentiality while utilizing RWD was regarded as a significant concern that needs to be addressed.  C. Data standardization and harmonization: The lack of standardized practices for data collection and reporting across healthcare institutions was recognized as a barrier. Workshop participants stressed the importance of implementing data standardization and harmonization efforts to enhance the usability of RWD for HTA purposes. |
| Makady A, et al, (2018) | Challenges include:  A. Differences in RWD nature impact melanoma prevalence data in REAs and long-term effectiveness in CEAs.  B. Appraisal of RWD often leans negatively due to concerns about biases and statistical approaches.  3) The lack of robust RWD at initial assessments, absence of systematic guidance, and logistical challenges with Pragmatic Clinical Trials (PCTs) contribute to the difficulties in RWD integration. |
| Deverka PA, et al, (2020) | Challenges and issues:  A. Lack of relevance and timeliness: Payers express concerns about RWE studies lacking relevance and timely information for coverage or utilization management decisions.  B. Perceived lack of rigor and transparency: Ratepayers may not consider the study methods used in RWE research as sufficiently rigorous and transparent.  C. Limited impact outside specific areas: There is a limited number of RWE studies beyond oncology, pharmacogenomics (PGx), and perinatal/pediatric examples that directly influence coverage decisions for NGS.  D. Additional training issues: Payers and clinicians may lack expertise in utilizing RWE and evaluating NGS testing.  E. Lack of structured genomic data in electronic health records (EHRs) hampers RWD/RWE analysis  F. Limited publicly available information on how RWE informs payer decision-making.  G. Inconsistent adherence to methodological guidance undermines evidence reliability. |
| Lou J, et al, (2020) | HTA dossier challenges:  A. Many respondents (9 out of 14) indicated that there was insufficient evidence to ensure the patient sample represents routine clinical care.  B. Few respondents (6 out of 14) mentioned the lack of information on other treatments and patient comorbidities.  C. Other reported challenges were long-term patient follow-up, inclusion of unrelated outcomes, and sample population similarity to clinical trials.  Infrastructure and Skills Challenges:  D. One major problem reported by stakeholders was the lack of infrastructure and skills for efficient RWD collection and utilization, including theabsence of HER electronic medical records in some Asian health systems.  E. Respondents also highlighted the lack of clinician, institution, or legislative support for data collection and sharing. |
| Facey KM, et al, (2020) | Fragmentation, lack of clarity, and harmonization challenges:  A. Fragmentation and divergent guidelines in RWD and RWE use in HTA.  B. Lack of clarity on suitable RWDs and robust RWE development with harmonizated approaches issues. C. Isolated approaches and insufficient collaboration among stakeholders. |
| Bowrin K, et al, (2019) | The limitations encountered in the integration of RWD into modeling are as follows:  A. Confounding bias and bias from selection  B. Lack of data accuracy regarding drug exposure and outcomes, as derived from the use of pharmacoepidemiological data  C. Errors in the data recording process of medical records  D. Inadequate assurance of data protection and confidentiality, which arises in cases of RWD utilization from medical records E. Insufficient patient numbers. |
| Brogaard N, et al, (2021) | A. Variation in requirements and evidence criteria: Different HTA organizations internationally have different requirements for evidence and criteria for evaluating treatments regardless of indication. This variation can lead to mixed assessment outcomes and challenges regarding reimbursement. From the analysis of 13 submissions from seven countries, different clinical evidence requirements were identified by HTA organizations in the evaluations of larotrectinib and entrectinib.  B. Uncertainty regarding clinical data: Payers express concerns about clinical data, especially when non-traditional datasets such as RWD are used. This uncertainty can contribute to negative or partial funding recommendations in certain countries.  C. Limited integration of RWD: The use of RWD in HTA evaluations is relatively low. The limited acceptance of RWD by some HTA organizations or the availability of RWD during the evaluation process can contribute to this. Further guidance is needed to clarify the acceptance of RWD as a data source for treatments regardless of indication and acceptable methods of indirect comparisons. |
| Hogervorst Milou A,  et al, (2022) | Several significant barriers include:  A. Lack of necessary data sources: One of the obstacles is the unavailability or insufficient access to the required data sources for conducting robust RWD studies.  B. Existing policy structures or information governance: The presence of rigid policy structures or inadequate information governance frameworks can hinder the integration and utilization of RWD.  C. Interpretation and verification challenges: Difficulties may arise in interpreting and verifying the results obtained from RWD studies, leading to uncertainty or lack of confidence in the findings.  D. Absence of relevant variables in registries: In some cases, registries may lack essential variables or data elements necessary for comprehensive analysis, limiting the potential insights that can be derived from the RWD.  E. Lengthy data access process: Delays or lengthy procedures to access the required data can impede timely utilization of RWD, affecting the efficiency and effectiveness of decision-making processes. |
| Sievers H, et al, (2021) | Challenges of RWE include:  A. Methodological issues: This encompasses concerns such as selection bias, difficulty in accessing relevant data, and the lower degree of evidence compared to Randomized Controlled Trials (RCTs).  B. Lower data quality: RWE may have a lower degree of data quality when compared to data derived from RCTs. This can be attributed to various factors, including variations in data collection methodologies and potential limitations in data accuracy.  C. Limited infrastructure for collecting RWD: There is a lack of well-established and standardized systems for collecting high-quality RWD. This poses challenges in ensuring the reliability and consistency of the data used for analysis.  D. Representativeness of RWD data: RWD may not always fully represent the broader population or specific subgroups of interest. This can limit the generalizability of findings and raise concerns about the applicability of RWE in decision-making processes. |
| Hampson G, et al, (2018) | Challenges in Utilizing RWE:  Α. Bias and Confounding Factors  Β. Incomplete Data Availability  C. Data Accessibility Challenges  D. Absence of Consensus on Methodologies for Design, Collection, and Analysis of Data  E. Shortage of Qualified Researchers |
| George E, (2016) | Issues in the Use of Non-Randomized Controlled Trials (non-RCTs):  A. Confusion and Bias: The use of non-RCTs can lead to confusion and bias in the interpretation of results. Factors such as selection bias and confounding variables can impact the validity and reliability of the findings.  B. Incomplete Data and Limited Use of Quality of Life Data: Non-RCTs often lack comprehensive data and may have limited utilization of quality of life data from sources outside of RCTs. This limitation can affect the ability to capture relevant information and adequately assess patient-reported outcomes.  C. Uncertainty in Data Accuracy: There is inherent uncertainty in the accuracy of data collected through non-RCTs. The lack of rigorous randomization and control measures may introduce additional variability, making it challenging to ascertain the true effect of interventions. |
| Husereau, D, et al, (2019) | The barriers to increasing the use of RWD are as follows:  1. Lack of trust in RWD due to concerns about data quality and reliability.  2. Difficulty in accessing RWD due to privacy and security concerns or regulatory and legal barriers that restrict access.  3. Lack of standardization and harmonization of RWD, as data may be collected using different methods and standards, making it challenging to compare data from different sources or jurisdictions.  4. Insufficient infrastructure to support the use of RWD, such as funding for data collection and analysis or a lack of expertise in data analysis.  5. Perceived threat to vested interests, as some stakeholders may feel that the use of RWD threatens their interests or can be used against them.  6. Lack of collaboration and coordination among stakeholders, as successful use of RWD requires collaboration and partnerships among stakeholders.  7. Need for education and training to increase understanding of RWD and their decision-making capabilities.  8. Difficulty in translating RWD into actionable insights, as the data may be complex or require advanced analytical tools. |
| Pongiglione B,  et al, (2021) | Barriers identified include:  (i) Difficulty in accessing data.  (ii) Lack of standardization in measuring health and economic outcomes.  (iii) Insufficient availability of comparable data.  (iv) Concerns about data quality and accuracy in certain sources.  (v) Inadequate presence of demographic and epidemiological data in some sources. |
| Ciminata, G, (2019) | The challenges associated with utilizing RWD to evaluate direct oral anticoagulation (DOAC) in the atrial fibrillation population include:  1) Limited availability of high-quality RWD: There is a scarcity of reliable and comprehensive RWD specifically for evaluating DOACs in the atrial fibrillation population. This limitation hinders the ability to obtain robust evidence for assessing the effectiveness and safety of DOACs.  2) Data quality issues: Challenges arise from incomplete data, inconsistent coding practices, and limited clinical information within the available datasets. These issues may compromise the reliability and validity of the results derived from the RWD.  3) Methodological challenges: The utilization of RWD for HTA purposes is subject to methodological challenges, including potential selection bias, confounding factors, and misclassification of outcomes. These challenges can impact the accuracy and interpretation of the results obtained from the analysis of RWD.  4) Data access issues: Accessing RWD can be challenging due to concerns regarding privacy, data ownership, and regulatory barriers. These factors may restrict the use of RWD in HTA and impede the ability to derive meaningful insights from the data. |

RCT: Randomized Clinical Trials, RWD: Real-World Data, HTA: Health Technology Assessment, ERG: Evidence Review Group, NICE: National Institute for Health and Care, Excellence, G-BA: Gemeinsamer Bundesausschuss (Federal Joint Committee, Germany), CADTH: Canadian Agency for Drugs and Technologies in Health, PBAC: Pharmaceutical Benefits Advisory Committee (Australia), AMNOG: Arzneimittelmarkt-Neuordnungsgesetz (German Pharmaceutical Market Reorganisation Act), GSAV: Gesetz für mehr Sicherheit in der Arzneimittelversorgung (Law for Greater Security in Drug Supply, Germany), SMC: Scottish Medicines Consortium, IQWiG: Institut für Qualität und Wirtschaftlichkeit im Gesundheitswesen (Institute for Quality and Efficiency in Healthcare, Germany), ZIN: Zorginstituut Nederland (National Health Care Institute, Netherlands), AIFA: Agenzia Italiana del Farmaco (Italian Medicines Agency), ICER: Incremental Cost-Effectiveness Ratio, EC: External Comparator, FDA: Food and Drug Administration, HER: Electronic Health Record, CEA: Cost-Effectiveness Analysis, REA: Relative Effectiveness Assessment, RWE: Real-World Evidence, RCTs: Randomized Controlled Trials, TAVI: transcatheter aortic valve implantation, TMVR: transcatheter mitral valve repair, DOAC: Direct Oral Anticoagulation
