# Supplementary material for: Real-world data: a comprehensive literature review on the barriers, challenges, and opportunities associated with their inclusion in the health technology assessment process
Source: J Pharm Pharm Sci. 2024 Feb 28;27:12302. doi: 10.3389/jpps.2024.12302 (PMC10932954; doi:10.3389/jpps.2024.12302)
Supplement: Supplementary file 6 [file Table4.docx]

**Table 4. Potential Benefits, Opportunities, and Feasibility of Utilizing RWD/RWE in the HTA Process**

| **Author/year** | **Potential Benefits, Opportunities, and Feasibility** |
| --- | --- |
| Hagen G, et al (2021) | Efficient Registries for Comprehensive Data: Well-structured patient registries provide valuable data on health utilities, resource utilization, and costs relevant to HTA procedures. These registries play a crucial role in understanding current treatment patterns, adherence, and the natural progression of diseases, especially in real-world scenarios. Recommended for economic evaluation modeling, patient registries serve as additional evidence, validating real-world clinical effectiveness, particularly in oncology.  Collaboration and Future Utilization: Collaboration between epidemiologists and HTA researchers is needed for more effective integration of data from population studies and registries. The data obtained from registries is anticipated to significantly contribute to health technology reassessments in the HTA process, with expectations of substantial advancements in their utilization in the coming years. |
| Gonçalves E, (2020) | Role of RWD in Advanced Therapies Assessment: Efficiently collecting and analyzing RWD should complement the evaluation of long-term efficacy and comparative effectiveness of advanced therapies.  Addressing Data Gaps with Outcome Modeling: Emphasizing outcome modeling offers a crucial opportunity to handle indirect comparisons between different treatments. This approach can effectively bridge gaps arising from the absence of appropriate short-term clinical trial and RWE data. |
| Fasseeh A, et al. (2020) | Investment in Patient Registries and Local Data Utilization: A significant majority of respondents (82%) expressed their willingness to invest in patient registries and make payer databases accessible to HTA professionals This initiative, especially in technologically advanced healthcare systems, serves as a valuable learning experience for HTA providers, contributing to improved assessment quality.  Reinforcing Local Data Significance in Policy Decisions: In policy decision-making, there is a need to reinforce the significance of local documentation and local RWD, which can be achieved by extensively utilizing local patient registries and payer databases. |
| Leahy T P, et al. (2020) | Role of Primary Care Databases in Healthcare Decision-Making: Primary care databases are playing an increasingly important role in informing NICE submissions and showcasing the utilization of RWE in healthcare decision-making. The inclusion of primary care databases to inform clinical inputs in cost-effectiveness (CE) models is well received by both the Evidence Review Group (ERG) and the review committees, particularly when a dedicated study is conducted to extract these inputs. The absence of extensive commentary observed when databases were utilized to inform current treatment practices may indicate a general acceptance that databases serve as reliable sources of such data. The potential of UK primary care databases in NICE submissions is being increasingly recognized and utilized, particularly in informing the parameters of economic models. |
| Fuchs S, et al (2016) | Initiatives Addressing RWE Challenges: Several initiatives have been undertaken to tackle these challenges. These initiatives include:  A. The implementation of standardized data elements and outcome measures  B. the advancement of analytical methods and tools for analyzing RWE  C. the establishment of data exchange platforms and networks. RWD and evidence from non-RCTs are progressively gaining recognition for HTA of medical devices in Europe. |
| Dai WF, et al, (2021) | Stakeholder Recognition of RWE Impact: The participating stakeholders acknowledged that utilizing Real-World Evidence (RWE) enhances confidence in clinical benefit and cost-effectiveness results across provinces.. They stressed the value of this evidence, especially in supporting policy decisions during the re-evaluation process. When considering RWE from other areas, adherence to accepted standards and bias management is crucial, with greater acceptance noted for rare diseases due to challenges in solely relying on Canadian data.  An additional highlight was the active inclusion of patient experience in reassessment processes, emphasizing patient-generated outcomes (PROs). Stakeholders proposed engaging the same patients from the initial evaluation during the re-evaluation of oncology drugs. |
| Patel D, et al, (2021) | Evaluating evidence-based submissions for HTA based on single-arm trials lacks formal guidance. Constructing comparison cohorts is challenging, necessitating a well-designed strategy. This involves robust methods for data collection and a sound analytical approach for indirect treatment comparison.  Monitoring and guideline development: The increasing utilization of RWD in HTA submissions, albeit still relatively low (5% of all submissions), indicates a growing trend. It is important to closely monitor this category of submissions, and the study can assist HTA providers and payers in developing guidelines and policies for effectively handling such submissions.  Oncology, rare diseases, and orphan drugs: Orphan drugs have a higher acceptance rate, with reliable External Comparators (ECs) emphasized for non-orphan drugs. RWD-based ECs show positive results, particularly in single-arm trials focusing on oncology and hemato-oncology indications. The period between 2015 and 2019 saw increased use and acceptance of RWD-based ECs, especially noted by NICE. |
| Tolley, K. (2010) | Observational Studies and Changing Access Strategies: Observational studies, including registries, gain importance post-marketing, particularly with conditional reimbursement schemes in countries like the Netherlands.  Certain countries, like the Netherlands, have implemented conditional drug reimbursement schemes, where reimbursement is provided temporarily for expensive and innovative medicines, with subsequent provision of evidence-based RWE. A similar case was observed in the UK regarding multiple sclerosis. As a result, drug access strategies are being formulated, which will eventually lead to increased demand for data beyond traditional clinical trials. In light of these developments, companies should consider adapting their research and development strategies accordingly.  Need for Diverse Study Approaches: Traditional RCTs alone may not suffice for comprehensive health technology evaluation. Pragmatic trials and observational studies, offering higher external validity, become crucial for assessing cost-effectiveness relationships in the context of drug access and reimbursement. |
| Kent, S. et al, (2021) | When data from RCTs are insufficient for making informed decisions, it is crucial to implement rigorous and comprehensive procedures to ensure the quality of data derived from non-randomized studies. Recommendations for addressing the issues encountered in conducting studies:  A) Provide justification for the need to conduct the study and highlight the use of non-randomized study data.  B) Design prospective studies and engage in early scientific advisory processes.  C) Understand potential systematic errors and provide clear references to mitigation and management strategies.  D) Perform sensitivity analyses for hypothetical scenarios.  E) Pre-specify protocols with clear references to statistical methods.  Z) Provide comprehensive reporting of methods and results, with the additional use of checklists.  H) Offer quantitative evidence when identifying uncertainty.  Θ) Describe impartiality and utilize well-validated tools for assessing overall risk.  Recommendations for addressing issues in HTA:  A) Enhance and standardize the provision of scientific advisory processes to determine the value of specific evidence. B) Strengthen conditional reimbursement processes to ensure the production of further evidence after initial reimbursement decisions.  C) Invest in and develop skills of HTA agency personnel in the design, analysis, and interpretation of non-randomized studies.  D) Issue guidelines on best practices.  E) Support further research on non-randomized studies and international initiatives for high-quality data. |
| Jaksa A, et al, (2022) | Evaluation of External Control Arms (ECAs): While the organizations did not reach a consensus on the quality and impact of external control arms (ECAs), two main criticisms were identified: selection bias and confusion. These concerns can be addressed through careful data selection and study design, aiming to minimize bias and improve clarity. Integrating Real-World Data (RWD) and Real-World Evidence (RWE) in Health Technology Assessment (HTA) complements single-arm oncology trials, offering contextual information on patient experience for a more comprehensive understanding of treatment outcomes and perspectives. Ongoing exploration beyond oncology is crucial to understand ECAs' influence across therapeutic areas.Guidance and Criticisms of ECAs: Selection bias and confounding emerged as common criticisms of ECAs, emphasizing the importance of utilizing high-quality RWD and implementing rigorous study designs. There is a clear need for additional guidance on study design and implementation when utilizing ECAs, as well as specific guidance on RWD-based ECAs from regulatory bodies such as the FDA. |
| Justo N, et al, (2019) | Balancing Local and International RWE: The HTA organizations must strike a balance between promoting local RWE without excessively delaying assessments. International RWE can be considered, addressing transferability concerns. With expanding healthcare coverage, RWE becomes vital for monitoring outcomes and cost-effectiveness of high-cost interventions, necessitating careful monitoring.  Enhancing RWD Usability: Initiatives improving data recording, including electronic medical records (EMRs), standardized coding systems, and staff training, present opportunities to enhance RWD usability.  Establishment of RWD units and evidence-based policy: The increasing number of HTA units and the adoption of pharmacoeconomic guidelines and evidence-based policy planning are encouraging trends for the utilization of RWE. These advancements offer a promising outlook for the expanded use of RWE in South America. |
| Kamusheva M, et al, (2022) | Adequate management of collected RWE from other countries is crucial, and the electronic collection of RWE is necessary. However, some Central and Eastern European countries face challenges due to their limited operational information technology infrastructure. Furthermore, the lack of government stability and frequent policy changes in the healthcare sector result in regularly amended and unstable legislation. The dynamic environment leads to constant changes in local legislative requirements for HTA and significant differences in approaches to managing the process of RWE utilization. Therefore, achieving stability is essential in this regard.  Moreover, collaborative initiatives and infrastructure development are expected to increase to facilitate the transferability of RWE to CEE countries. The focus will shift towards determining the appropriate implementation of RWE. Lastly, there is a need for improvement in factors such as pharmacotherapy guidelines and the availability of health technologies in certain CEE countries. Additionally, the development of suitable statistical analysis methods for the transmission of RWE is urgently required. |
| Timbie JW, et al, (2021) | Recommendations for addressing the issues related to the inclusion of RWE:  1) Provide more specific and up-to-date guidance on the utilization of RWE, including successful examples of its application. Industry stakeholders emphasize the need for clear guidance from payers, which would assist in designing studies using RWD.  2) Enhance the robustness of RWE by improving study design and analytical methods. This would ensure that the research conducted using RWD meets rigorous standards and produces reliable and valid results.  3) Engage in RWE-related activities sponsored by organizations like NESTcc, CDRH, and industries, which bring together stakeholders to develop a framework for enhancing RWE generation and detection methods.  4) Take part in the FDA's Payor Communication Task Force and share insights gained from working group discussions. This collaboration allows the industry to gather important information for designing studies using RWD, while payers gain a deeper understanding of reimbursement decisions in studies utilizing RWD.  5) Develop alternative reimbursement strategies supported by RWE when sufficient experimental data is not available to support full coverage. Some payers are exploring alternative payment arrangements that are tied to the performance of medical devices in real-world settings, providing an avenue for reimbursement based on RWE outcomes. |
| Bullement A, et al, (2020) | Optimizing RWE Utilization in NICE Submissions: Companies seeking to enhance the utilization of Real-World Evidence (RWE) in submissions to the National Institute for Health and Care Excellence (NICE) should proactively address common criticisms. This involves justifying RWE analysis methodologies and demonstrating the relevance and reliability of RWE sources. Developing best practice guidelines for reporting Real-World Data (RWD) and consistently adhering to these standards can significantly improve the effectiveness of documentation submitted to NICE.  Significance of RWE in Decision-Making: RWE has demonstrated its significance in decision-making processes by filling data gaps in cost-effectiveness analyses submitted by companies. This data is ultimately utilized to inform decisions, particularly through its impact on the ICER estimate. This study indicates that the utilization of RWE in NICE submissions for cancer drugs is widespread and generally proves to be a valuable source of information, effectively supporting the decision-making process. |
| Al-Omar HA, et al, (2021) | In relation to the challenges associated with RWD in the assessment, treatment, and evaluation (process, the following observations were made:  Availability and accuracy of data: The availability and accuracy of data, including registries, RWE, quality of life (QoL), and costing data, were deemed important by 86.4% of participants.  Scattered healthcare system in Saudi Arabia: The fragmented nature of the healthcare system in Saudi Arabia was recognized as a challenge, which could impact the collection, integration, and accessibility of reliable RWD.  Lack of acceptance by stakeholders: There was a lack of acceptance and recognition of the value of RWD by various stakeholders involved in the HTA process, which may hinder its effective utilization.  Lack of expertise and human resources: Insufficient expertise and limited human resources in the field of RWD were identified as challenges, potentially impeding the effective analysis and interpretation of available data.  Lack of reliable data on diseases: The absence of reliable and comprehensive data on specific diseases was highlighted as a challenge, which may limit the applicability and generalizability of RWD in the HTA process. |
| Makady A, et al, (2018) | Alignment with policies: The findings of this study are in line with a previous review of policies regarding the use of RWD by HTA organizations. The review discovered that the current utilization of RWD in practice is consistent with the policies, which may vary slightly between agencies and depending on the specific context being analyzed.  Pragmatic type trials (PCTs) as a potential source of RWD: PCTs were suggested as a potential means of gathering RWD that can bridge the gap between randomized controlled trials (RCTs) and real-world settings.  Quantitative methods and sensitivity analyses: The study discusses quantitative modeling and sensitivity analysis methods, such as bootstrapping and probabilistic sensitivity analyses (PSA), as potential alternatives to RWD for addressing the efficacy-effectiveness gap. However, it should be noted that these methods are still based on hypotheses and RCT data, and the importance of RWD remains crucial for evaluating long-term efficacy in a diverse clinical population.  Potential role in re-assessments: RWDs could play a significant role in the reassessment of drugs by confirming previous efficacy estimates, cost-effectiveness ratios (ICERs), and budget implications. Comprehensive re-evaluation reports conducted under conditional reimbursement systems (CRS) could greatly benefit from the utilization of RWDs. |
| Deverka PA, et al, (2020) | Opportunities for training and development incentives in RWE and NGS: A. Offer training programs on observational study methods and pragmatic type trials (PCTs) to stakeholders, including payers, to enhance their understanding and utilization of RWE.  B. Encourage funding initiatives to support the conduct and publication of RWE studies that demonstrate the benefits and risks associated with NGS testing. This would provide valuable evidence to inform coverage decisions.  Opportunities for improving methods of generating RWE:  A. Existing RWE assessment tools developed specifically for payers should be adapted to the context of NGS through the collaboration of multi-stakeholder groups. This would ensure best practices and evaluation tools are tailored to the unique aspects of NGS.  B. Develop transparent engagement processes that involve payers to better understand their information needs and incorporate them into the design and execution of RWE studies.  C. Additionally, the use of artificial intelligence (AI)-based methods, such as natural language processing, machine learning, and deep learning, can be leveraged to process and analyze unstructured data from electronic health records (EHRs) and patient-generated data. This would alleviate the burden of manual curation and enhance the efficiency of data analysis in RWE studies.  Despite the potential benefits, payers still heavily rely on clinical guidelines and randomized controlled trials (RCTs) as the primary sources of clinical evidence for coverage decisions, rather than RWE. Several factors contribute to this reliance, including challenges surrounding the quality of RWE data, standardization of genomic data representation, limited involvement of payers in study development, and a preference for RCTs over observational data. Efforts are underway to address these barriers, such as the development of data infrastructure, increased payer participation in study design, adherence to methodological best practices, and consensus on NGS guidelines.  Outcomes-based contracts (OBCs) between industry and payers can benefit from the utilization of RWE. However, challenges related to obtaining accurate data, defining appropriate outcome measures, ensuring patient data privacy, and managing costs often impede the widespread implementation of OBCs. |
| Lou J, et al, (2020) | Guidance Document for Asia: The development of a guidance document specifically tailored to the context of Asia can be instrumental in addressing the challenges identified in this study. This guidance document can provide recommendations, best practices, and standardized approaches for the utilization of RWD and RWE in the region, thereby supporting stakeholders in navigating the complexities and mitigating the identified challenges.  Collaboration for Overcoming Challenges: Collaboration among various stakeholders, including healthcare providers, researchers, policymakers, and industry representatives, is crucial in overcoming the challenges associated with RWD/RWE utilization. By fostering collaboration and promoting the adoption of good practices, stakeholders can share knowledge, experiences, and expertise, leading to improved methodologies, data quality, and overall effectiveness in utilizing RWD/RWE.  Supplementary role of RWE in HTA Process: All participants reached a consensus that RWE should serve as supplementary documentation and is unlikely to replace data derived from clinical trials for reimbursement decisions. The findings suggest a recognition of the potential of RWD/RWE in HTA, along with a need for further understanding, capacity-building, and improved collaboration among stakeholders to fully harness the benefits of utilizing RWD/RWE in the HTA process. |
| Facey KM, et al, (2020) | Opportunities and applications:  A. Collaborating with the academic community to develop methods for utilizing RWD in the decision-making process, as well as promoting cooperation between countries, especially in the context of rare diseases.  B. Involving the industry in multilateral dialogues regarding RWD, through initiatives such as public-private partnerships and international programs like the IMI Big Data for Better Outcomes in Europe and the US FDA's Sentinel program.  C. Governance and transparency: Ensuring transparency in the production of RWE is crucial. Efforts are being made to address this issue through initiatives like the ISPOR RWE Transparency Task Force, which aims to promote transparency in the generation and reporting of RWE.  D. EU Multilateral Learning Network for RWE: Establishing a learning network that involves relevant stakeholders is essential for harnessing the potential of RWD and RWE in healthcare. This network should focus on data standardization, improving data quality, facilitating data sharing, and implementing robust data processing and analysis methods.  E. Regulatory support: Regulatory agencies, such as the US FDA, are actively engaged in RWE projects aimed at providing guidance on the relevance and reliability of RWD. These initiatives involve leadership engagement and target shared knowledge and consistency in regulatory approaches. They are widely recognized as necessary measures to address the challenges associated with RWD/RWE and ensure their effective utilization in regulatory decision-making processes. |
| Bowrin K, et al, (2019) | Optimizing Registry Data for Cost-Effectiveness Modeling: To overcome limitations in modeling data through registries, a practical guide recommends using registry data effectively for evidence-based decisions on the cost-effectiveness of new drugs. Additionally, evaluating cost-effectiveness models with observational data can be facilitated by employing a specialized checklist, featuring five questions to assess statistical issues.  Utilization of RWE in Clinical Practice:  Ten guidelines from organizations emphasize the usefulness of Real-World Evidence (RWE) in providing evidence for clinical practice, covering treatment pathways, comparative interventions, resource utilization, costs, long-term disease progression, and the actual effectiveness and safety of interventions.. Sixteen (16) pharmacoeconomic guidelines recommend or consider the integration of RWE into pharmacoeconomic models and HTA submissions, with Canada, Belgium, and Poland recommending the incorporation of RWD into HTA submissions. |
| Brogaard N, et al, (2021) | Enhancing RWD Acceptance for Tumor-Agnostic Therapies: Monitoring future assessments and potential reassessments would provide a better acceptance of RWD as a data source for tumor-agnostic therapies, and further guidance is needed to clarify what is acceptable regarding indirect comparisons.  Conditional reimbursement: Some countries, such as England and France, have established conditional approval processes. This allows treatments with data uncertainty to receive funding recommendations while requiring the provision of additional data at a later date. This provides an opportunity for the acceptance of currently available evidence, including RWD/RWE, with the potential for further data collection and evaluation.  Lack of guidance for HTA evaluations: There is a need for more guidance on how HTA evaluations and economic models should be structured for future oncology-agnostic therapies. Some HTA systems are developing internal guidelines to ensure consistent assessment of oncology-agnostic therapies, but further guidance is still needed to address uncertainties in clinical data and the integration of RWD. |
| Hogervorst Milou A, et al, (2022) | RWD could be considered acceptable in the following situations:  A. In cases of high disease burden or when the indications of the evaluated treatment are severe or even life-threatening.  B. If the findings of RCTs are outdated or conflicting with the available literature of RCTs.  C. When there is uncertainty regarding resource utilization in clinical practice.  D. In the case of highly innovative health technologies or when alternative access is not feasible.  E. When the trials used for licensing are compared with treatments not used in the country's practice.  F. In situations of lack of reliable evidence and when there is uncertainty in the clinical domain as well as a high degree of uncertainty in cost-effectiveness analyses.  G. In cases of well-established use, according to European legislation. |
| Sievers H, et al, (2021) | Capabilities of RWE include:  A. Filling gaps and reducing uncertainty: RWE can provide supplementary documentation that supports the data obtained from Randomized Controlled Trials (RCTs), helping to address any gaps or uncertainties in the evidence base.  B. Demonstrating real-world value: RWE allows for the evaluation of a medicine's effectiveness and value in everyday clinical practice, providing insights into its performance outside the controlled environment of clinical trials.  C. Ethical considerations: In situations where conducting an RCT may be ethically challenging or not feasible, RWE offers an opportunity to gather evidence and generate insights.  Opportunities associated with RWE include: Harmonization and greater acceptance:  A. RWE presents an opportunity for harmonizing documentation requirements and increasing the acceptance of RWE as a valuable source of evidence in healthcare decision-making processes.  B. Stakeholder dialogue and consensus: Through plHTAorms like the Joint Scientific Advice, stakeholders can engage in discussions and reach consensus on various issues, bridging the gaps between regulatory requirements and the expectations of HTA agencies.  C. Early joint dialogues: Particularly during the stages when the European Medicines Agency (EMA) is considering technology licensing, early joint dialogues involving multiple stakeholders can help facilitate a more comprehensive understanding of the evidence landscape.  D. Alignment of HTA documentation: The EUnetHTA plHTAorm provides an opportunity for aligning documentation requirements between different HTAs, promoting consistency and efficiency in the evaluation of healthcare technologies. |
| Hampson G, et al, (2018) | Opportunities for Improving the Use of RWE:  A. Enhancing the quality and reliability of RWE studies through the establishment of a national mandatory registry for observational studies, national data repositories, investments in the quality and consistency of electronic medical records, and the development of strict protocols and consensus on best practice guidelines.  B. Implementing effective governance regulations that clarify the scope of RWE data that can be shared.  C. Greater commitment to the development of pragmatic trial designs, which can bridge the evidence gap between RWE and Randomized Controlled Trials (RCTs).  D. Fast-track approval processes, such as accelerated approval pathways, for various innovations where sufficient evidence is lacking. In these processes, RWE can play a decisive role. |
| George E, (2016) | Opportunities for Improving Non-Randomized Controlled Trials (non-RCTs):  Α. International Collaboration: The participation of NICE in cross-border projects, such as the IMI Get Real project and the ADAPT SMART project, can contribute to the development of frameworks and methodologies for integrating RWD into HTA processes.  Β. Areas for Improvement: There are opportunities to enhance the usability and reliability of non-RCT data. This includes improvements in case identification and data source linkage, the routine capture of health-related quality of life (HRQL) and patient preferences, the inclusion of important patient variables (sociodemographic, disease severity, comorbidities), and explicit variable definitions to minimize data gaps.  C. Complementary Use of RCTs and RWD: RCTs alone cannot address all the needs of HTA and decision-makers. The utilization of data from non-RCT sources provides an opportunity to strengthen evidence standards and generate evidence throughout the technology lifecycle. RCTs and RWD can complement each other to provide a more comprehensive understanding of effectiveness and safety in healthcare interventions. |
| Makady E, et al, (2017) | Harmonizing RWD Policies for Enhanced Utilization: The lack of alignment and guidance regarding the practical aspects of RWD collection and analysis may discourage marketing authorization holders from investing in RWD generation for HTA purposes. Clarity and harmonization of policies could provide incentives for stakeholders to collect and utilize RWD more effectively.  The approach of policy alignment regarding the use of RWD by HTA organizations in Europe is useful, and the presentation of guidelines for the collection and analysis of such data is recommended. Policy harmonization can appropriately support the industry in producing additional or alternative data for drugs where RCTs cannot provide strong evidence. EUnetHTA can support harmonization through a discussion plHTAorm. The use of RWD can be particularly valuable for rare diseases or orphan drugs where conducting randomized controlled trials (RCTs) may be challenging. RWD can provide data on treatment outcomes in situations where conventional clinical trials are not feasible. |
| Husereau, D, et al, (2019) | Future Growth of RWD Utilization through various initiatives: The use of RWD is expected to increase in the future, supported by technological advancements, the growing availability of data, and the increasing demand for evidence to support decision-making. To fully harness the potential of RWD, there is a need for continuous investment in data infrastructure, including standardization and harmonization of data, development of analytical tools, and personnel training.  Enhancing Collaboration for RWD Potential: There is a need for greater collaboration and partnerships among stakeholders, including researchers, regulatory authorities, payers, and industry, to ensure the quality and reliability of RWD and promote their use in decision-making. These opportunities highlight the potential for leveraging RWD to generate RWE that can inform healthcare policies, improve patient outcomes, and enhance the efficiency and effectiveness of healthcare interventions. By addressing the challenges and investing in the necessary infrastructure and collaborations, RWD can be a valuable resource in shaping the future of healthcare. |
| Pongiglione B, et al, (2021) | Ensuring Standardization for Reliable RWD: Ensuring standardization is essential, encompassing consistent and appropriate selection, measurement, utilization, and reporting of results in clinical research and practice. This approach will aid in mitigating biases and enhancing the validity and reliability of findings. Enhanced coordination at the European Union (EU) level would be particularly advantageous in facilitating the generation of comparable RWD across countries.  Advancing RWD Utilization through Collaboration: To further advance the utilization of RWD, a collaborative approach involving stakeholders is necessary for the initiation, design, and analysis of RWD, with industry cooperation. If discussions at the EU level regarding early HTA and the requirement for registers or observation studies become more prevalent, the quality and nature of RWD collected will assume increasing importance in informing policy decisions. |
| Ciminata, G, (2019) | Standardizing RWD Processes: There is a need to standardize the collection, management, and analysis of RWD in order to enhance the quality and comparability of the results. By incorporating RWD, cost-effectiveness analyses can inform HTA decisions regarding the utilization of DOACs versus warfarin in individuals with atrial fibrillation. |

HTA: Health Technology Assessment, RWD: Real-World Data, RWE: Real-World Evidence, NICE: National Institute for Health and Care Excellence, ECs: External Comparators, FDA: U.S. Food and Drug Administration, EMRs: Electronic Medical Records, CEE: Central and Eastern European, NESTcc: National Evaluation System for health Technology Coordinating Center, CDRH: Center for Devices and Radiological Health, ICER: Incremental Cost-Effectiveness Ratio, EMA: European Medicines Agency, IMI: Innovative Medicines Initiative, RCTs: Randomized Controlled Trials, HRQL: Health-Related Quality of Life, EU: European Union, DOACs: Direct Oral Anticoagulants
