# Supplementary material for: Real-world data: a comprehensive literature review on the barriers, challenges, and opportunities associated with their inclusion in the health technology assessment process
Source: J Pharm Pharm Sci. 2024 Feb 28;27:12302. doi: 10.3389/jpps.2024.12302 (PMC10932954; doi:10.3389/jpps.2024.12302)
Supplement: Supplementary file 3 [file DataSheet1.ZIP › Supplementary Material/S2 Appendix. Quality assessment of included studies.zip!/Quality assessment of included studies/Checklist_for_Systematic_Reviews_and_Research_Syntheses_Leahy T P, et al, (2020) .pdf]

CHECKLIST FOR SYSTEMATIC REVIEWS AND RESEARCH SYNTHESES

Critical Appraisal tools for use in JBI Systematic Reviews

jbi.global

CRICOS Provider Number 00123M

INTRODUCTION

JBI is an international research organisation based in the Faculty of Health and Medical Sciences at the University of Adelaide, South Australia. JBI develops and delivers unique evidence-based information, software, education and training designed to improve healthcare practice and health outcomes. With over 70 Collaborating Entities, servicing over 90 countries, JBI is a recognised global leader in evidence-based healthcare.

JBI Systematic Reviews

The core of evidence synthesis is the systematic review of literature of a particular intervention, condition or issue. The systematic review is essentially an analysis of the available literature (that is, evidence) and a judgment of the effectiveness or otherwise of a practice, involving a series of complex steps. JBI takes a particular view on what counts as evidence and the methods utilised to synthesise those different types of evidence. In line with this broader view of evidence, JBI has developed theories, methodologies and rigorous processes for the critical appraisal and synthesis of these diverse forms of evidence in order to aid in clinical decision-making in healthcare. There now exists JBI guidance for conducting reviews of effectiveness research, qualitative research, prevalence/incidence, etiology/risk, economic evaluations, text/opinion, diagnostic test accuracy, mixed-methods, umbrella reviews and scoping reviews. Further information regarding JBI systematic reviews can be found in the JBI Evidence Synthesis Manual.

JBI Critical Appraisal Tools

All systematic reviews incorporate a process of critique or appraisal of the research evidence. The purpose of this appraisal is to assess the methodological quality of a study and to determine the extent to which a study has addressed the possibility of bias in its design, conduct and analysis. All papers selected for inclusion in the systematic review (that is – those that meet the inclusion criteria described in the protocol) need to be subjected to rigorous appraisal by two critical appraisers. The results of this appraisal can then be used to inform synthesis and interpretation of the results of the study. JBI Critical appraisal tools have been developed by the JBI and collaborators and approved by the JBI Scientific Committee following extensive peer review. Although designed for use in systematic reviews, JBI critical appraisal tools can also be used when creating Critically Appraised Topics (CAT), in journal clubs and as an educational tool.

© JBI, 2020. All rights reserved. JBI grants use of these tools for research purposes only. All other enquiries should be sent to jbisynthesis@adelaide.edu.au.

JBI CRITICAL APPRAISAL CHECKLIST FOR SYSTEMATIC REVIEWS AND RESEARCH SYNTHESES

Reviewer: Konstantinos Zisis Date: 11 May 2023

Author: Leahy T P, et al. Year: 2020 Record Number: N/A

		Yes	No	Unclear	Not applicable
1.	Is the review question clearly and explicitly stated?	✓	☐	☐	☐
2.	Were the inclusion criteria appropriate for the review question?	✓	☐	☐	☐
3.	Was the search strategy appropriate?	✓	☐	☐	☐
4.	Were the sources and resources used to search for studies adequate?	✓	☐	☐	☐
5.	Were the criteria for appraising studies appropriate?	☐	☐	✓	☐
6.	Was critical appraisal conducted by two or more reviewers independently?	☐	✓	☐	☐
7.	Were there methods to minimize errors in data extraction?	☐	✓	☐	☐
8.	Were the methods used to combine studies appropriate?	✓	☐	☐	☐
9.	Was the likelihood of publication bias assessed?	☐	✓	☐	☐
10.	Were recommendations for policy and/or practice supported by the reported data?	✓	☐	☐	☐
11.	Were the specific directives for new research appropriate?	☐	☐	✓	☐

Overall appraisal: Include ✓ Exclude ☐ Seek further info ☐

Comments (Including reason for exclusion)

The findings of this study suggest that when planning Health Economics and Outcomes Research (HEOR) strategies to support market access in England, greater consideration should be given to the inclusion and purposeful utilization of UK primary care databases in NICE submissions. This can help enhance the quality and relevance of real-world evidence in the technology assessment process. This study is of moderate quality.

© JBI, 2020. All rights reserved. JBI grants use of these tools for research purposes only. All other enquiries should be sent to jbisynthesis@adelaide.edu.au.

JBI CRITICAL APPRAISAL CHECKLIST FOR SYSTEMATIC REVIEWS AND RESEARCH SYNTHESIS

REFERENCES

1. Whiting P, Rutjes AWS, Reitsma JB, Bossuyt PMM, Kleijnen J. The development of QUADAS: a tool for the quality assessment of studies of diagnostic accuracy included in systematic reviews. BMC Medical Research Methodology. 2003;3:25 doi:10.1186/1471-2288-3-25.

© JBI, 2020. All rights reserved. JBI grants use of these tools for research purposes only. All other enquiries should be sent to jbisynthesis@adelaide.edu.au.
