# Supplementary material for: Real-world data: a comprehensive literature review on the barriers, challenges, and opportunities associated with their inclusion in the health technology assessment process
Source: J Pharm Pharm Sci. 2024 Feb 28;27:12302. doi: 10.3389/jpps.2024.12302 (PMC10932954; doi:10.3389/jpps.2024.12302)
Supplement: Supplementary file 3 [file DataSheet1.ZIP › Supplementary Material/S2 Appendix. Quality assessment of included studies.zip!/Quality assessment of included studies/Checklist_for_Text_and_Opinion_Tolley (2010).pdf]

CHECKLIST FOR TEXT AND OPINION

Critical Appraisal tools for use in JBI Systematic Reviews

jbi.global

CRICOS Provider Number 00123M

INTRODUCTION

JBI is an international research organisation based in the Faculty of Health and Medical Sciences at the University of Adelaide, South Australia. JBI develops and delivers unique evidence-based information, software, education and training designed to improve healthcare practice and health outcomes. With over 70 Collaborating Entities, servicing over 90 countries, JBI is a recognised global leader in evidence-based healthcare.

JBI Systematic Reviews

The core of evidence synthesis is the systematic review of literature of a particular intervention, condition or issue. The systematic review is essentially an analysis of the available literature (that is, evidence) and a judgment of the effectiveness or otherwise of a practice, involving a series of complex steps. JBI takes a particular view on what counts as evidence and the methods utilised to synthesise those different types of evidence. In line with this broader view of evidence, JBI has developed theories, methodologies and rigorous processes for the critical appraisal and synthesis of these diverse forms of evidence in order to aid in clinical decision-making in healthcare. There now exists JBI guidance for conducting reviews of effectiveness research, qualitative research, prevalence/incidence, etiology/risk, economic evaluations, text/opinion, diagnostic test accuracy, mixed-methods, umbrella reviews and scoping reviews. Further information regarding JBI systematic reviews can be found in the JBI Evidence Synthesis Manual.

JBI Critical Appraisal Tools

All systematic reviews incorporate a process of critique or appraisal of the research evidence. The purpose of this appraisal is to assess the methodological quality of a study and to determine the extent to which a study has addressed the possibility of bias in its design, conduct and analysis. All papers selected for inclusion in the systematic review (that is – those that meet the inclusion criteria described in the protocol) need to be subjected to rigorous appraisal by two critical appraisers. The results of this appraisal can then be used to inform synthesis and interpretation of the results of the study. JBI Critical appraisal tools have been developed by the JBI and collaborators and approved by the JBI Scientific Committee following extensive peer review. Although designed for use in systematic reviews, JBI critical appraisal tools can also be used when creating Critically Appraised Topics (CAT), in journal clubs and as an educational tool.

© JBI, 2020. All rights reserved. JBI grants use of these tools for research purposes only. All other enquiries should be sent to jbisynthesis@adelaide.edu.au.

JBI CRITICAL APPRAISAL CHECKLIST FOR TEXT AND OPINION PAPERS

Reviewer: Konstantinos Zisis Date: 09 May 2023

Author: Tolley K Year: 2010 Record Number: N/A

	Yes	No	Unclear	Not applicable
1. Is the source of the opinion clearly identified?	√	☐	☐	☐
2. Does the source of opinion have standing in the field of expertise?	☐	☐	√	☐
3. Are the interests of the relevant population the central focus of the opinion?	√	☐	☐	☐
4. Is the stated position the result of an analytical process, and is there logic in the opinion expressed?	√	☐	☐	☐
5. Is there reference to the extant literature?	√	☐	☐	☐
6. Is any incongruence with the literature/sources logically defended?	☐	☐	√	☐

Overall appraisal: Include √ Exclude ☐ Seek further info ☐

Comments (Including reason for exclusion)

This commentary appears to be of good quality with few unclear points.

© JBI, 2020. All rights reserved. JBI grants use of these tools for research purposes only. All other enquiries should be sent to jbisynthesis@adelaide.edu.au.

EXPLANATION OF TEXT AND EXPERT OPINION CRITICAL APPRAISAL TOOL

How to cite: *McArthur A, Klugarova J, Yan H, Florescu S. Innovations in the systematic review of text and opinion. Int J Evid Based Healthc. 2015;13(3):188–195.*

Answers: Yes, No, Unclear or Not/Applicable

1. **Is the source of the opinion clearly identified?**

   Is there a named author? Unnamed editorial pieces in journals or newspapers, or magazines give broader licence for comment, however authorship should be identifiable.

2. **Does the source of opinion have standing in the field of expertise?**

   The qualifications, current appointment and current affiliations with specific groups need to be stated in the publication and the reviewer needs to be satisfied that the author(s) has some standing within the field.

3. **Are the interests of the relevant population the central focus of the opinion?**

   The aim of this question is to establish the author's purpose in writing the paper by considering the intended audience. If the review topic is related to a clinical intervention, or aspect of health care delivery, a focus on health outcomes will be pertinent to the review. However, if for example the review is focused on addressing an issue of inter-professional behaviour or power relations, a focus on the relevant groups is desired and applicable. Therefore this question should be answered in context with the purpose of the review.

4. **Is the stated position the result of an analytical process, and is there logic in the opinion expressed?**

   In order to establish the clarity or otherwise of the rationale or basis for the opinion, give consideration to the direction of the main lines of argument. Questions to pose of each textual paper include: What are the main points in the conclusions or recommendations? What arguments does the author use to support the main points? Is the argument logical? Have important terms been clearly defined? Do the arguments support the main points?

5. **Is there reference to the extant literature?**

   If there is reference to the extant literature, is it a non-biased, inclusive representation, or is it a non-critical description of content specifically supportive of the line of argument being put forward? These considerations will highlight the robustness of how cited literature was managed.

6. **Is any incongruence with the literature/sources logically defended?**

   Is there any reference provided in the text to ascertain if the opinion expressed has wider support? Consider also if the author demonstrated awareness of alternate or dominant opinions in the literature and provided an informed defence of their position as it relates to other or similar discourses.

© JBI, 2020. All rights reserved. JBI grants use of these tools for research purposes only. All other enquiries should be sent to jbisynthesis@adelaide.edu.au.
